# Hepatic steatosis is associated with postprandial dysglycemia in type 1 diabetes: insights from dual-modality liver assessment

**DOI:** 10.3389/fmed.2026.1933235

**Published:** 2026-10-27

**Authors:** Shuang Li, Panzun Wang, Zhihong Cai, Tianyu Gao, Qihang Chen, Yiwen Zhou, Mingdan Gu, Yanjun Jin, Min Shen, Xinyu Xu, Yun Shi, Tao Yang, Yang Chen, Yong Gu

**Affiliations:** 1 Department of Endocrinology and Metabolism, The First Affiliated Hospital with Nanjing Medical University, Nanjing, China; 2 Department of Science and Education, Kunshan Sixth People's Hospital, Suzhou, Jiangsu, China

**Keywords:** 1, 5-anhydroglucitol, continuous glucose monitoring, insulin resistance, insulin-like growth factor-1, metabolic dysfunction-associated steatotic liver disease, type 1 diabetes

## Abstract

**Background:**

Metabolic dysfunction-associated steatotic liver disease (MASLD) is increasingly recognized in type 1 diabetes (T1D), yet its relationship with dynamic glycemic abnormalities and the metabolic pathways underlying these associations remains incompletely understood. We investigated the associations of hepatic steatosis with conventional and continuous glucose monitoring (CGM)-derived glycemic measures in T1D and explored the potential roles of circulating insulin-like growth factor-1 (IGF-1) and insulin resistance.

**Methods:**

This cross-sectional study included 231 individuals with T1D who underwent hepatic steatosis assessment using ultrasonography and controlled attenuation parameter (CAP). Glycemic status was characterized using glycated hemoglobin (HbA1c), fasting plasma glucose, 1,5-anhydroglucitol (1,5-AG), and, in 142 participants, at least 72 h of valid CGM under standardized inpatient conditions. Multivariable linear regression, false discovery rate correction, and exploratory serial mediation analyses were performed.

**Results:**

Ultrasound-defined MASLD was present in 50 participants (21.6%). After multivariable adjustment, ultrasound-defined MASLD and greater CAP were consistently associated with lower 1,5-AG concentrations and less favorable CGM profiles, including higher 2-hour postprandial glucose, glucose peak, mean glucose, glucose management indicator, and time above range, together with lower time in range, whereas fasting glucose was not significantly associated with hepatic steatosis. Hepatic steatosis was also associated with lower circulating IGF-1 concentrations and greater insulin resistance across multiple surrogate indices. In exploratory serial mediation analysis using TG/HDL-C as the primary insulin resistance measure, the combined indirect association through IGF-1 and insulin resistance accounted for 82.6% of the association between MASLD and lower 1,5-AG concentrations, with consistent patterns observed using alternative insulin resistance indices.

**Conclusions:**

Hepatic steatosis in T1D is independently associated with a distinct dysglycemic phenotype characterized particularly by short-term and postprandial hyperglycemia. Concurrent differences in circulating IGF-1 and insulin sensitivity may statistically account for a substantial proportion of this association. These findings support hepatic steatosis as a clinically relevant metabolic comorbidity in T1D and warrant prospective studies to clarify the underlying temporal and biological relationships.

## Graphical Abstract

**Figure:**
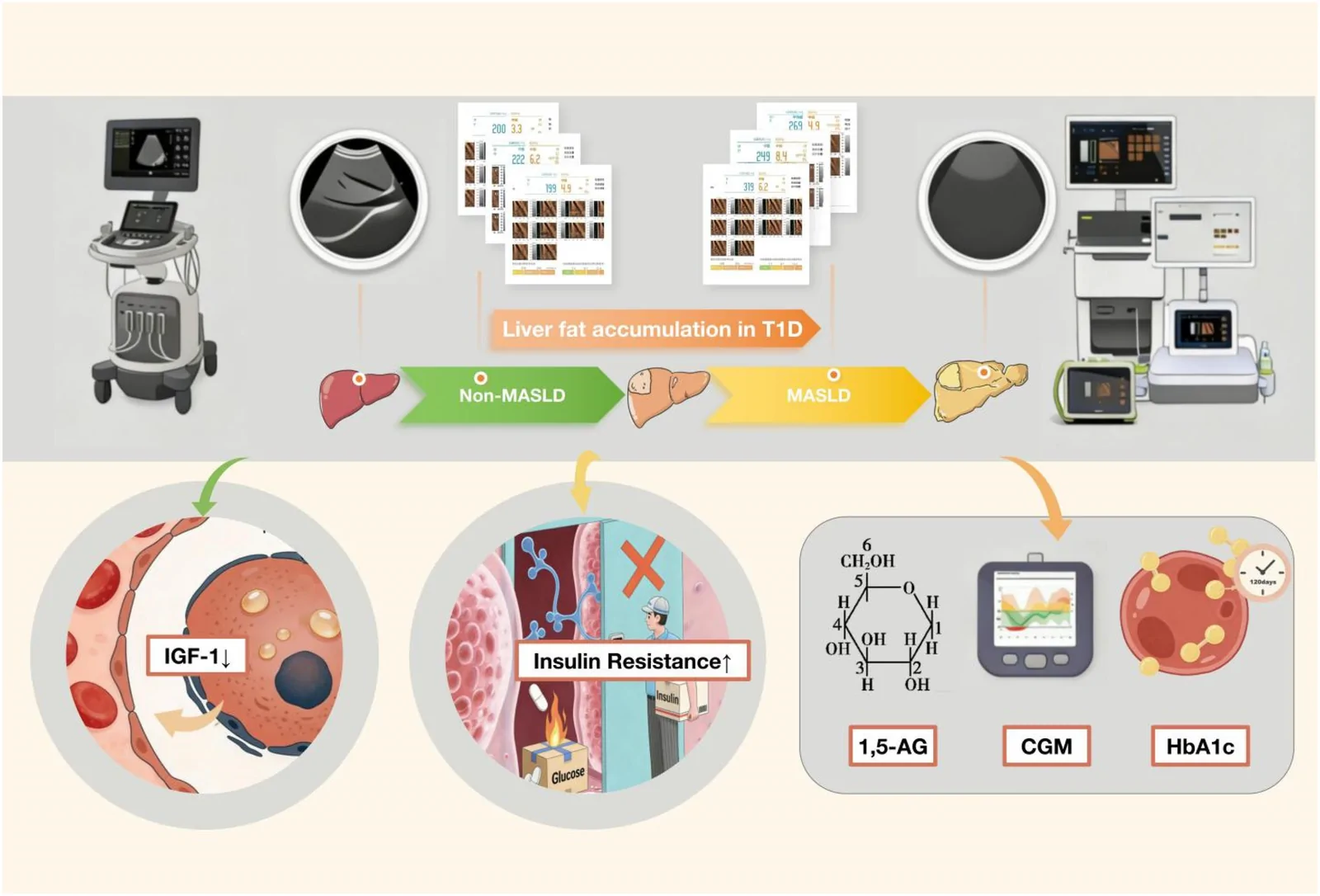

## 1 Introduction

Metabolic dysfunction-associated steatotic liver disease (MASLD) has become one of the most prevalent chronic liver disorders worldwide, affecting nearly 40% of the adult population and representing an increasingly important cause of liver-related morbidity and mortality (1, 2). Its close association with type 2 diabetes (T2D) is well established, with insulin resistance serving as a central pathophysiological link between hepatic steatosis and dysglycemia (3). In contrast, the prevalence, metabolic consequences, and clinical significance of MASLD in type 1 diabetes (T1D) remain incompletely understood.

Although insulin resistance is a central driver of hepatic steatosis in T2D, the pathophysiological context may differ in T1D. In addition to the increasing contribution of obesity and insulin resistance, T1D-specific factors such as nonphysiological exogenous insulin delivery, altered insulin clearance, chronic hyperglycemia, dysregulated glucagon signaling, and reduced glucagon-like peptide-1 concentration may uniquely influence hepatic glucose and lipid metabolism (4, 5). These differences suggest that the relationship between hepatic steatosis and glycemic regulation in T1D should be investigated within its distinct metabolic context rather than directly extrapolated from T2D. At the same time, the growing prevalence of overweight and obesity among individuals with T1D has contributed to the emergence of a “double diabetes” phenotype characterized by the coexistence of autoimmune insulin deficiency, excess adiposity, insulin resistance, and increased cardiometabolic risk (6). Intensive insulin therapy, defensive eating behaviors aimed at preventing or treating hypoglycemia, and increasingly sedentary lifestyles may further contribute to this metabolic transition (7). Consequently, hepatic steatosis is increasingly recognized as an important metabolic comorbidity in T1D and may be associated with diabetic complications, chronic kidney disease, and adverse clinical outcomes (8, 9).

Despite growing interest in this field, the glycemic phenotype associated with MASLD in T1D remains insufficiently characterized. Previous studies have largely focused on the prevalence of hepatic steatosis and its associations with conventional metabolic risk factors or surrogate measures of insulin resistance (10, 11). Glycemic assessment has frequently relied on glycated hemoglobin (HbA1c) or fasting glucose, which may not adequately capture postprandial hyperglycemia and short-term glucose fluctuations. Although continuous glucose monitoring (CGM) enables more comprehensive characterization of dynamic glucose patterns, evidence linking MASLD with CGM-derived glycemic profiles in T1D remains limited. Furthermore, many previous studies were conducted under free-living conditions, in which variations in dietary intake, physical activity, and insulin administration may contribute to residual confounding. Few studies have combined complementary assessments of hepatic steatosis with both biochemical markers and CGM-derived metrics under standardized conditions (12, 13).

The biological mechanisms potentially linking hepatic steatosis to dysglycemia in T1D also remain poorly defined. The liver is the principal source of circulating insulin-like growth factor-1 (IGF-1), a hormone that plays an important role in glucose homeostasis and insulin sensitivity. Experimental and clinical studies have shown that hepatic steatosis is associated with impairment of the growth hormone (GH)/IGF-1 axis and reduced circulating IGF-1 concentrations (14–16). In parallel, MASLD has been associated with increased insulin resistance, including among individuals with T1D (17–19). These pathways may not operate independently, as reduced IGF-1 availability may itself contribute to impaired insulin sensitivity, suggesting a potential framework linking hepatic steatosis, IGF-1 dysregulation, insulin resistance, and abnormal glucose regulation (20, 21). However, the relative contributions of these pathways to glycemic dysregulation in T1D remain unclear.

Therefore, we aimed to comprehensively characterize the glycemic phenotype associated with hepatic steatosis in individuals with T1D using two complementary assessments of liver fat burden, namely ultrasound-defined MASLD and controlled attenuation parameter (CAP), together with biochemical and CGM-derived glycemic measures under standardized inpatient conditions. We further examined whether liver fat burden was independently associated with glycemic dysregulation and explored whether alterations in circulating IGF-1 concentrations and insulin resistance-related indices statistically accounted for these associations.

## 2 Methods

### 2.1 Study design and participants

This cross-sectional study investigated the associations between hepatic steatosis, glycemic control, circulating IGF-1, and insulin resistance in individuals with T1D. Participants were consecutively recruited from the Department of Endocrinology, The First Affiliated Hospital of Nanjing Medical University, between November 2024 and February 2026. The study protocol was approved by the institutional ethics committee (Approval No. 2025-SR-291), and all participants provided written informed consent before enrollment. The study was conducted in accordance with the Declaration of Helsinki and Good Clinical Practice guidelines.

Eligible participants were required to have a diagnosis of T1D based on biochemical evidence of diabetes together with at least one positive pancreatic islet autoantibody (IAA, ICA, GADA, IA-2A, or ZnT8A), or a typical clinical history consistent with T1D as determined by an experienced diabetologist (22). Additional inclusion criteria were a stable treatment regimen for at least three months before enrollment, willingness to undergo liver imaging assessments, and availability of complete clinical and laboratory data.

During the study period, a total of 461 individuals with T1D were screened for eligibility. Among them, 230 were excluded according to the prespecified exclusion criteria, including alternative causes of hepatic steatosis, excessive alcohol consumption, pregnancy or lactation, severe renal impairment [estimated glomerular filtration rate (eGFR) < 30 mL/min/1.73 m^2^], recent use of medications known to affect hepatic steatosis or glucose metabolism, inflammatory bowel disease, active malignancy, previous pancreatic resection or major gastrointestinal surgery, anemia, diabetic ketoacidosis, severe hypoglycemia, acute illness within four weeks before enrollment, or self-reported irregular dietary habits. Ultimately, 231 participants fulfilled the eligibility criteria and were included in the primary analyses. The detailed participant screening and enrollment process is presented in Supplementary Figure 1.

All 231 participants underwent liver imaging and laboratory assessments. CGM was additionally offered to participants during the study period and participation was voluntary. Among the 231 enrolled participants, 142 agreed to undergo CGM and completed at least 72 h of valid glucose recording, whereas 89 declined CGM participation. The 142 participants with valid CGM data were included in analyses involving CGM-derived metrics.

### 2.2 Standardized metabolic stabilization protocol

To minimize the influence of short-term metabolic fluctuations and lifestyle-related confounding, all participants underwent a standardized 3-day inpatient stabilization protocol before study assessments.

During this period, participants received a standardized diabetic diet prepared according to institutional nutritional guidelines and engaged only in supervised low-intensity physical activity. Glycemic management was standardized using continuous subcutaneous insulin infusion (MiniMed Paradigm 712; Medtronic, Northridge, CA, USA). Individualized basal insulin rates and meal-related bolus doses were optimized by study endocrinologists and recorded throughout the stabilization period.

Following an overnight fast of at least 10 h, participants underwent anthropometric measurements, venous blood sampling, abdominal ultrasonography, and vibration-controlled transient elastography (VCTE). For participants undergoing CGM, glucose monitoring was performed under the same standardized dietary, physical activity, and insulin-management conditions.

### 2.3 Laboratory assessments and definitions

After completion of the stabilization protocol and an overnight fast, venous blood samples were collected for biochemical analyses.

Fasting plasma glucose was measured using an automated enzymatic assay (Beckman Coulter, USA). HbA1c was measured by ion-exchange high-performance liquid chromatography (D-100, Bio-Rad Laboratories). Serum 1,5-anhydroglucitol (1,5-AG), a biomarker of short-term hyperglycemic excursions, was quantified using a commercial enzymatic assay (GlycoMark™). Lipid parameters, including total cholesterol, triglycerides, low-density lipoprotein cholesterol (LDL-C), and high-density lipoprotein cholesterol (HDL-C), together with liver enzymes, were analyzed using an automated biochemical analyzer (AU5800, Beckman Coulter). Fasting C-peptide concentrations were quantified using a chemiluminescent immunoassay (Roche Diagnostics, Switzerland). Values below the lower limit of detection were imputed as the lower limit of detection divided by √2 (23). Renal function was assessed using the eGFR calculated according to the Modification of Diet in Renal Disease equation (24). Diabetes-associated autoantibodies were assessed as follows: GADA, ZnT8A, and IA-2A were measured using standardized radiobinding assays. IAA was determined using a commercial enzyme-linked immunosorbent assay, whereas ICA was detected by indirect immunofluorescence (25, 26).

Insulin resistance and sensitivity were assessed estimated using the estimated glucose disposal rate (27) (eGDR), the insulin sensitivity index of Search (28) (ISI-Search), TG-glucose (29) (TyG) index and TG to HDL-C ratio (28) (TG/HDL-C).

$$\begin{array}{c} \mathrm{eGDR}=21.158\;-\;0.090\;\times \;\mathrm{waist}\;\mathrm{circumference}(\mathrm{cm})\;-\;3.407\;\\ \times \;\mathrm{hypertension}\;\mathrm{status}\;(1=\mathrm{yes};\;\;0=\mathrm{no})\;-\;0.551\;\;\;\\ \times \;\mathrm{HbA}1c\;(\%)\mathrm{ISI}-\mathrm{SEARCH}=\exp[4.64725-0.02032\;\\ \times \mathrm{waistcircumference}(\mathrm{cm})-0.09779\;\\ \times \mathrm{HbA}1c(\mathrm{TyG}=\mathrm{Ln}\;[\mathrm{fasting}\;\mathrm{TGs}\;(\mathrm{mg}/\mathrm{dL})\;\\ \times \;\mathrm{fasting}\;\mathrm{glucose}\;(\mathrm{mg}/\mathrm{dL})\;/\;2]\mathrm{TG}/\mathrm{HDL}-C\;\\ =\mathrm{TG}\;(\mathrm{mg}/\mathrm{dL})\;/\;\mathrm{HDL}-C\;(\mathrm{mg}/\mathrm{dL})\;\; \end{array}$$

### 2.4 Hepatic steatosis quantification

Liver fat was assessed using two complementary imaging approaches after completion of the stabilization protocol and overnight fasting. Ultrasound-defined hepatic steatosis was used as the primary categorical exposure, whereas CAP was analyzed as a continuous measure of liver fat burden in the primary association analyses.

First, conventional B-mode abdominal ultrasonography was performed by experienced radiologists who were blinded to participants' clinical and laboratory data. Hepatic steatosis was diagnosed when at least two of the following sonographic features were present: increased hepatorenal echogenicity, vascular blurring, and posterior beam attenuation (30). In participants fulfilling the cardiometabolic criteria for MASLD, ultrasound-defined hepatic steatosis was considered compatible with MASLD after exclusion of excessive alcohol intake and other secondary causes of hepatic steatosis.

Second, all participants underwent VCTE (FibroScan® Pro; Echosens, Paris, France) to obtain measurements of CAP and liver stiffness (31). CAP was analyzed as a continuous measure of liver fat burden. Quality criteria required at least 10 valid measurements and an interquartile range-to-median ratio <30% (32).

For sensitivity analyses, MASLD was alternatively defined using a CAP threshold of ≥238 dB/m, based on previously published CAP thresholds for hepatic steatosis (33, 34).

### 2.5 Assessment of glycemic control

Glycemic control was evaluated using complementary biochemical and CGM-derived metrics. Biochemical measures included HbA1c, fasting plasma glucose, and serum 1,5-AG. HbA1c reflects long-term glycemic exposure, whereas 1,5-AG provides additional information regarding recent hyperglycemic excursions and postprandial glucose exposure.

A subset of participants underwent blinded CGM using the Yuwell CT2 system. Participants were required to complete at least 72 h of valid glucose recording under standardized dietary and physical activity conditions. CGM-derived metrics included time in range (TIR, 3.9–10.0 mmol/L), time above range (TAR, >10.0 mmol/L), mean glucose (MG), glucose management indicator (GMI), glucose peak, glucose nadir, and 2-hour postprandial glucose (PPG-2 h).

Insulin doses administered during CGM recording were documented and incorporated as covariates in multivariable analyses.

### 2.6 Statistical analysis

Continuous variables are presented as mean ± standard deviation (SD) or median [interquartile range (IQR)], as appropriate, whereas categorical variables are presented as frequencies and percentages. Between-group comparisons were performed using Student's *t*-test, the Mann–Whitney *U*-test, the *χ*^2^ test, or Fisher's exact test, as appropriate.

Associations between hepatic steatosis and metabolic outcomes were examined using multivariable linear regression models. Hepatic steatosis was analyzed both as a categorical variable (ultrasound-defined MASLD) and as a continuous variable using CAP. Glycemic outcomes included HbA1c, fasting plasma glucose, 1,5-AG, and CGM-derived metrics. For continuous outcomes with skewed distributions, natural log transformation was performed before analysis.

Three sequential regression models were constructed. Model 1 was unadjusted. Model 2 adjusted for demographic, clinical, metabolic, and lifestyle-related factors, including age, sex, diabetes duration, body mass index (BMI), daily insulin dose per kilogram of body weight, fasting C-peptide concentration, hypertension, lipid-lowering medication use, eGFR, current smoking status and alcohol consumption. Model 3 further adjusted for diabetes-related autoimmune characteristics, including GADA, IA-2A, IAA, ICA, and ZnT8A positivity. Model 3 was prespecified as the primary fully adjusted model, with covariates selected *a priori* based on biological plausibility and previous literature rather than data-driven statistical criteria. Associations of hepatic steatosis with circulating IGF-1 concentrations and surrogate measures of insulin resistance were evaluated using the same modeling strategy.

To facilitate comparison of association magnitudes across outcomes measured on different scales, standardized beta coefficients (St.*β*) with corresponding 95% confidence intervals (CIs) were reported as the primary effect estimates. Continuous exposure and outcome variables were standardized to z-scores before regression analysis. For analyses involving the binary ultrasound-defined MASLD exposure, the exposure was retained as a categorical variable while the continuous outcome was standardized, such that the coefficient represents the difference in the outcome in SD units between participants with and without MASLD. For analyses involving continuous CAP, unstandardized regression coefficients were additionally reported per 10 dB/m increase in CAP to provide clinically interpretable effect estimates. To account for multiple comparisons among prespecified glycemic and insulin resistance outcomes, false discovery rate (FDR) correction using the Benjamini–Hochberg procedure was performed for the primary fully adjusted Model 3 analyses. FDR-adjusted *P*-values were reported as sensitivity analyses to evaluate the robustness of the observed associations.

Prespecified subgroup analyses were conducted according to age (≤30 vs. >30 years), diabetes duration (≤10 vs. >10 years), sex, BMI (<24 vs. ≥24 kg/m^2^), and autoantibody status. Potential effect modification was evaluated by including multiplicative interaction terms in fully adjusted models, with interaction *P*-values considered the primary evidence for heterogeneity.

Given the cross-sectional design, serial mediation analyses were performed to explore whether concurrent alterations in circulating IGF-1 and insulin resistance statistically accounted for the association between hepatic steatosis and glycemic dysregulation. These analyses were intended to quantify potential explanatory pathways and should not be interpreted as evidence of causality. Mediation models were estimated using PROCESS macro-Model 6 (version 4.1) with 5,000 bootstrap resamples to generate bias-corrected 95% CIs. Serum 1,5-AG was selected as the primary glycemic outcome because it is particularly sensitive to recent hyperglycemic excursions and postprandial glucose exposure. TG/HDL-C was selected as the primary surrogate measure of insulin resistance because its calculation is independent of contemporaneous glucose concentrations, thereby reducing potential collinearity with glycemic outcomes. Sensitivity analyses were performed by replacing TG/HDL-C with the TyG index, eGDR, and ISI-Search.

All statistical analyses were conducted using IBM SPSS Statistics version 28.0 (IBM Corp., Armonk, NY, USA). All tests were two-sided. Statistical significance was defined as *P* < 0.05 for primary analyses, whereas FDR-adjusted *P*-values were interpreted in the multiple-comparison sensitivity analyses.

## 3 Results

### 3.1 Clinical characteristics of participants according to MASLD status

Among the 231 participants with T1D included in the study, 50 (21.6%) were diagnosed with MASLD by ultrasonography. Compared with participants without MASLD, those with MASLD were older and had a less favorable cardiometabolic profile, including higher BMI, greater waist circumference, higher triglyceride concentrations, and lower HDL-C levels (Table 1). CAP values were significantly higher in the MASLD group, whereas liver stiffness measurements did not differ significantly between groups.

**Table 1:** Baseline Characteristics According to Ultrasound-Defined MASLD Status

Variable	Overall (*N* = 231)	Non-MASLD (*n* = 181)	MASLD (*n* = 50)	*P*-value
Demographics				
Age, years	42.000 [23.833, 57.000]	40.000 [22.000, 56.000]	45.000 [33.000, 61.000]	0.045
Female sex, *n* (%)	118 (51.1%)	88 (48.6%)	30 (60.0%)	0.206
Anthropometric and clinical characteristics				
BMI, kg/m^2^	22.105 [20.200, 24.000]	21.640 [20.000, 23.600]	23.323 [21.400, 24.970]	0.003
Waist circumference, cm	79.000 [72.000, 86.000]	78.000 [71.000, 85.000]	83.000 [74.000, 92.000]	<0.001
Hypertension, *n* (%)	52 (22.5%)	38 (21.0%)	14 (28.0%)	0.391
Diabetes characteristics				
Age at diagnosis, years	33.001 [16.923, 49.491]	30.185 [15.335, 48.022]	38.764 [24.896, 50.751]	0.083
Diabetes duration, years	5.002 [1.224, 12.003]	5.002 [1.388, 12.000]	6.001 [0.331, 13.000]	0.842
Daily insulin dose, IU/kg	0.550 [0.345, 0.698]	0.550 [0.380, 0.706]	0.485 [0.039, 0.642]	0.108
Lifestyle factors				
Current smoker, *n* (%)	47 (20.3%)	37 (20.4%)	10 (20.0%)	>0.999
Current drinker, *n* (%)	35 (15.2%)	28 (15.5%)	7 (14.0%)	0.973
Liver and renal parameters				
CAP, dB/m	208.000 [180.000, 232.000]	199.000 [176.000, 219.000]	247.000 [229.000, 265.000]	<0.001
Liver stiffness, kPa	4.600 [3.900, 5.600]	4.600 [3.900, 5.400]	5.100 [4.000, 5.900]	0.122
ALT, U/L	15.000 [11.000, 21.000]	14.000 [11.000, 21.000]	15.000 [12.000, 20.000]	0.432
AST, U/L	19.000 [16.000, 24.000]	18.000 [16.000, 23.000]	19.000 [17.000, 27.000]	0.046
eGFR, mL/min/1.73m^2^	119.455 [99.639, 145.575]	119.203 [101.683, 145.575]	122.330 [91.785, 142.783]	0.771
Lipid Profile				
Total cholesterol, mmol/L	4.400 [3.720, 5.080]	4.360 [3.760, 5.080]	4.575 [3.360, 5.070]	0.996
Triglycerides, mmol/L	0.890 [0.620, 1.250]	0.800 [0.600, 1.110]	1.245 [0.880, 1.800]	<0.001
LDL cholesterol, mmol/L	2.590 [2.130, 3.150]	2.560 [2.180, 3.050]	2.895 [1.900, 3.290]	0.468
HDL cholesterol, mmol/L	1.300 [1.090, 1.530]	1.340 [1.130, 1.550]	1.150 [0.970, 1.360]	<0.001
Lipid-lowering Medication Use	23 (10.0%)	19 (10.5%)	4 (8.0%)	0.799
Insulin resistance/sensitivity indices				
eGDR	8.736 [6.968, 10.202]	8.986 [7.157, 10.545]	7.891 [6.411, 8.869]	0.001
ISI-Search	7.147 [5.529, 9.210]	7.957 [6.107, 9.840]	5.628 [4.374, 7.013]	<0.001
TyG	8.523 [8.039, 9.030]	8.431 [7.985, 8.910]	8.858 [8.343, 9.474]	<0.001
TG/HDL-C	1.625 [0.986, 2.502]	1.464 [0.923, 2.083]	2.493 [1.616, 3.964]	<0.001
*β*-cell Function				
Fasting C-peptide, pmol/L	57.600 [2.355, 244.800]	36.900 [2.355, 236.000]	129.150 [2.355, 244.800]	0.241
Autoantibody				
IAA positive, *n* (%)	64 (27.7%)	52 (28.7%)	12 (24.0%)	0.629
ICA positive, *n* (%)	47 (20.3%)	32 (17.7%)	15 (30.0%)	0.086
IA-2A positive, *n* (%)	76 (32.9%)	62 (34.3%)	14 (28.0%)	0.507
ZnT8A positive, *n* (%)	66 (28.6%)	54 (29.8%)	12 (24.0%)	0.528
GADA positive, *n* (%)	122 (52.8%)	88 (48.6%)	34 (68.0%)	0.023
Glycemic Control				
HbA1c, %	8.300 [6.790, 10.000]	8.070 [6.640, 9.930]	8.895 [7.900, 10.380]	0.006
Fasting glucose, mmol/L	7.400 [5.300, 9.410]	7.250 [5.280, 9.000]	7.590 [5.470, 10.360]	0.315
1,5-AG, μmol/L	22.230 [14.450, 37.870]	24.970 [14.990, 39.730]	17.210 [12.880, 29.310]	0.004
Circulating IGF-1				
IGF-1, ng/mL	125.000 [88.200, 164.000]	128.000 [98.400, 171.000]	97.750 [61.600, 160.000]	<0.001

Data are presented as median [Q1, Q3] for continuous variables and *n* (%) for categorical variables. *P* values were calculated using the Wilcoxon rank-sum test for continuous variables and the chi-square test or Fisher's exact test, as appropriate, for categorical variables.

1,5-AG, 1,5-anhydroglucitol; ALT, alanine aminotransferase; AST, aspartate aminotransferase; BMI, body mass index; CAP, controlled attenuation parameter; eGDR, estimated glucose disposal rate; eGFR, estimated glomerular filtration rate; GADA, glutamic acid decarboxylase antibody; HbA1c, glycated hemoglobin; HDL-C, high-density lipoprotein cholesterol; IAA, insulin autoantibody; IA-2A, protein tyrosine phosphatase-2 antibody; IGF-1, insulin-like growth factor-1; ISI-Search, insulin sensitivity index-Search; LDL-C, low-density lipoprotein cholesterol; MASLD, metabolic dysfunction-associated steatotic liver disease; TG/HDL-C, triglyceride-to-high-density lipoprotein cholesterol ratio; TyG, triglyceride-glucose index; ZnT8A, zinc transporter 8 antibody.

Participants with MASLD also exhibited a more insulin-resistant phenotype across all surrogate indices, characterized by lower eGDR and ISI-Search values and higher TyG and TG/HDL-C ratios (all *P* < 0.01). Circulating IGF-1 concentrations were also significantly lower in participants with MASLD than in those without MASLD (97.75 vs. 128.00 ng/mL, *P* < 0.001).

Regarding glycemic characteristics, participants with MASLD had higher HbA1c levels (8.90% vs. 8.07%, *P* = 0.006) and lower serum 1,5-AG concentrations (17.21 vs. 24.97 μmol/L, *P* = 0.004), whereas fasting plasma glucose did not differ significantly between groups. These findings indicate that participants with MASLD had a distinct metabolic phenotype characterized by greater insulin resistance, lower circulating IGF-1 concentrations, and less favorable long- and short-term glycemic markers.

Among the 231 participants, 142 agreed to undergo CGM and completed valid monitoring, whereas 89 declined CGM participation. Compared with participants who declined CGM, those included in the CGM subgroup were older and had higher HbA1c levels, lower 1,5-AG concentrations, greater insulin resistance, and lower circulating IGF-1 concentrations. CAP values and liver stiffness measurements did not differ significantly between the two groups (Supplementary Table 1).

### 3.2 Association of hepatic steatosis with glycemic control in T1D

Hepatic steatosis was associated with adverse glycemic profiles in Pearson correlation analyses (Supplementary Figure 2), and these associations generally persisted after multivariable adjustment (Table 2). In the fully adjusted Model 3, ultrasound-defined MASLD was associated with significantly lower serum 1,5-AG concentrations (St.*β* = −0.475, 95% CI −0.795 to −0.155, *P* = 0.004). Consistent results were observed for CAP as a continuous measure of liver fat burden, with each 10 dB/m increase in CAP associated with a 0.076-unit decrease in ln (1,5-AG) (*β* = −0.076, 95% CI −0.110 to −0.041, *P* < 0.001; St.*β* = −0.301). Ultrasound-defined MASLD was also associated with higher HbA1c levels (St.*β* = 0.467, 95% CI 0.136–0.797, *P* = 0.006), while each 10 dB/m increase in CAP was associated with a 0.012-unit increase in ln (HbA1c) (95% CI 0.001–0.023, *P* = 0.035; St.*β* = 0.158). In contrast, neither ultrasound-defined MASLD nor continuous CAP was significantly associated with fasting plasma glucose after full adjustment.

**Table 2:** Association between liver steatosis and glycemic profiles.

Outcome	Model	Ultrasound-defined MASLD		CAP, Continuous		
Venous Blood (*n* = 231)		St.*β* [95% CI], Yes or No	*P*-value	β [95% CI], per 10 dB/m	St.β [95% CI], per 1-SD	*P*-value
1,5-AG	Model 1	−0.419 [−0.729, −0.108]	0.008	−0.071 [−0.103, −0.040]	−0.284 [−0.408, −0.159]	<0.001
	Model 2	−0.427 [−0.743, −0.111]	0.008	−0.076 [−0.110, −0.041]	−0.301 [−0.439, −0.163]	<0.001
	Model 3	−0.475 [−0.795, −0.155]	0.004	−0.076 [−0.110, −0.041]	−0.301 [−0.438, −0.163]	<0.001
HbA1c	Model 1	0.401 [0.089, 0.712]	0.012	0.006 [−0.003, 0.016]	0.086 [−0.044, 0.215]	0.195
	Model 2	0.481 [0.158, 0.804]	0.004	0.012 [0.001, 0.023]	0.163 [0.017, 0.309]	0.029
	Model 3	0.467 [0.136, 0.797]	0.006	0.012 [0.001, 0.023]	0.158 [0.012, 0.304]	0.035
Fasting Glucose	Model 1	0.180 [−0.135, 0.494]	0.262	0.007 [−0.010, 0.024]	0.054 [−0.076, 0.184]	0.417
	Model 2	0.216 [−0.113, 0.546]	0.197	0.007 [−0.013, 0.026]	0.051 [−0.097, 0.199]	0.501
	Model 3	0.249 [−0.082, 0.581]	0.139	0.007 [−0.012, 0.026]	0.056 [−0.091, 0.202]	0.456
CGM profiles (*n* = 142)		St.β [95% CI], Yes or No	*P*-value	β [95% CI], per 10 dB/m	St.β [95% CI], per 1-SD	*P*-value
PPG-2 h	Model 1	0.714 [0.347, 1.082]	<0.001	0.195 [0.062, 0.328]	0.238 [0.076, 0.400]	0.004
	Model 2	0.675 [0.301, 1.049]	<0.001	0.165 [0.014, 0.315]	0.201 [0.017, 0.384]	0.033
	Model 3	0.714 [0.331, 1.096]	<0.001	0.189 [0.033, 0.345]	0.230 [0.040, 0.421]	0.018
Glucose Peak	Model 1	0.637 [0.266, 1.009]	<0.001	0.361 [0.130, 0.593]	0.252 [0.090, 0.413]	0.002
	Model 2	0.620 [0.231, 1.009]	0.002	0.326 [0.057, 0.594]	0.227 [0.040, 0.415]	0.018
	Model 3	0.609 [0.210, 1.009]	0.003	0.355 [0.077, 0.633]	0.248 [0.054, 0.442]	0.013
Glucose Nadir	Model 1	0.258 [−0.126, 0.642]	0.187	0.091 [0.006, 0.176]	0.177 [0.013, 0.341]	0.035
	Model 2	0.188 [−0.203, 0.578]	0.343	0.076 [−0.020, 0.171]	0.147 [−0.038, 0.331]	0.118
	Model 3	0.270 [−0.118, 0.657]	0.171	0.103 [0.008, 0.199]	0.200 [0.016, 0.384]	0.034
MG	Model 1	0.710 [0.342, 1.078]	<0.001	0.204 [0.090, 0.318]	0.286 [0.126, 0.446]	<0.001
	Model 2	0.671 [0.297, 1.044]	<0.001	0.186 [0.057, 0.314]	0.261 [0.080, 0.441]	0.005
	Model 3	0.709 [0.327, 1.092]	<0.001	0.215 [0.083, 0.347]	0.302 [0.116, 0.488]	0.002
GMI	Model 1	0.710 [0.342, 1.078]	<0.001	0.089 [0.039, 0.138]	0.288 [0.128, 0.448]	<0.001
	Model 2	0.671 [0.297, 1.044]	<0.001	0.081 [0.025, 0.136]	0.262 [0.082, 0.443]	0.005
	Model 3	0.709 [0.327, 1.092]	<0.001	0.093 [0.036, 0.151]	0.304 [0.118, 0.490]	0.002
TIR	Model 1	-0.639 [−1.010, −0.268]	<0.001	-2.219 [−3.493, −0.945]	-0.279 [−0.439, −0.119]	<0.001
	Model 2	-0.600 [−0.975, −0.225]	0.002	-2.109 [−3.533, −0.686]	-0.265 [−0.444, −0.086]	0.004
	Model 3	-0.647 [−1.032, −0.263]	0.001	-2.414 [−3.884, −0.943]	-0.304 [−0.489, −0.119]	0.001
TAR	Model 1	0.648 [0.278, 1.019]	<0.001	2.420 [1.086, 3.755]	0.290 [0.130, 0.449]	<0.001
	Model 2	0.608 [0.232, 0.985]	0.002	2.303 [0.805, 3.801]	0.276 [0.096, 0.455]	0.003
	Model 3	0.656 [0.271, 1.041]	<0.001	2.658 [1.115, 4.200]	0.318 [0.134, 0.503]	<0.001

Model 1: Crude model (unadjusted).

Model 2: Adjusted for age, sex, diabetes duration, body mass index, average daily insulin dose per kilogram, fasting C-peptide, hypertension, lipid-lowering medication use, eGFR, current smoking status, and current alcohol use.

Model 3: Additionally adjusted for GADA, IA-2A, IAA, ICA, and ZnT8A.

Values are standardized β coefficients (St.β) with 95% confidence intervals (CIs), unless otherwise specified. For ultrasound-defined MASLD, MASLD status was retained as a binary exposure, whereas continuous outcomes were standardized to z-scores; therefore, the St.β represents the difference in outcome standard deviation units between participants with and without MASLD. For continuous CAP analyses, unstandardized β coefficients per 10 dB/m increase in CAP and standardized β coefficients per 1-SD increase in CAP are presented. 1,5-AG, HbA1c, and fasting glucose were natural log-transformed before analysis.

1,5-AG, 1,5-anhydroglucitol; CAP, controlled attenuation parameter; CGM, continuous glucose monitoring; GADA, glutamic acid decarboxylase antibody; GMI, glucose management indicator; IAA, insulin autoantibody; IA-2A, protein tyrosine phosphatase-2 antibody; MASLD, metabolic dysfunction-associated steatotic liver disease; MG, mean glucose; PPG-2 h, 2-hour postprandial glucose; TAR, time above range; TIR, time in range; ZnT8A, zinc transporter 8 antibody.

Among the 142 participants with valid CGM data, hepatic steatosis was consistently associated with less favorable glucose profiles (Table 2). In Model 3, ultrasound-defined MASLD was associated with higher PPG-2 h (St.*β* = 0.714, 95% CI 0.331–1.096, *P* < 0.001), higher glucose peak (St.*β* = 0.609, 95% CI 0.210–1.009, *P* = 0.003), higher MG (St.*β* = 0.709, 95% CI 0.327–1.092, *P* < 0.001), and higher GMI (St.*β* = 0.709, 95% CI 0.327–1.092, *P* < 0.001). Ultrasound-defined MASLD was also associated with lower TIR (St.*β* = −0.647, 95% CI −1.032 to −0.263, *P* = 0.001) and higher TAR (St.*β* = 0.656, 95% CI 0.271–1.041, *P* < 0.001).

Similar associations were observed when hepatic fat burden was assessed continuously using CAP. Each 10 dB/m increase in CAP was associated with higher PPG-2 h (*β* = 0.189, 95% CI 0.033–0.345, *P* = 0.018), higher glucose peak (*β* = 0.355, 95% CI 0.077–0.633, *P* = 0.013), higher MG (*β* = 0.215, 95% CI 0.083–0.347, *P* = 0.002), and higher GMI (*β* = 0.093, 95% CI 0.036–0.151, *P* = 0.002). In addition, each 10 dB/m increase in CAP was associated with a 2.414-percentage-point reduction in TIR (95% CI −3.884 to −0.943, *P* = 0.001) and a 2.658-percentage-point increase in TAR (95% CI 1.115–4.200, *P* < 0.001). CAP was also positively associated with glucose nadir (*β* = 0.103, 95% CI 0.008 to 0.199, *P* = 0.034), although the corresponding association with ultrasound-defined MASLD was not statistically significant. Overall, the stronger and more consistent associations with 1,5-AG, PPG-2 h, and CGM-derived glucose profiles, together with the absence of a significant association with fasting glucose, suggest that hepatic steatosis is more prominently associated with postprandial and overall glycemic dysregulation than with fasting glycemia in individuals with T1D. These overall findings were largely preserved after Benjamini–Hochberg FDR correction of the fully adjusted Model 3 analyses (Supplementary Table 2).

Exploratory interaction analyses identified nominal effect modification by age, diabetes duration, IA-2A status, and ZnT8A status for the association with 1,5-AG (Supplementary Figure 3A and Supplementary Table 3), whereas ZnT8A status also modified the association with HbA1c. (Supplementary Figure 3B and Supplementary Table 4). Given the exploratory nature of these analyses and multiple subgroup comparisons, these findings should be interpreted cautiously.

### 3.3 Associations of hepatic steatosis with insulin resistance indices and circulating IGF-1

Hepatic steatosis was consistently associated with lower circulating IGF-1 concentrations and a more insulin-resistant metabolic profile (Table 3). In the fully adjusted Model 3, ultrasound-defined MASLD was associated with significantly lower circulating IGF-1 concentrations (St.*β* = −0.451, 95% CI −0.693 to −0.208, *P* < 0.001). MASLD was also associated with higher TG/HDL-C ratios (St.*β* = 0.859, 95% CI 0.574–1.145, *P* < 0.001) and higher TyG indices (St.*β* = 0.694, 95% CI 0.386–1.002, *P* < 0.001), together with lower eGDR (St.*β* = −0.348, 95% CI −0.561 to −0.135, *P* = 0.001) and ISI-Search values (St.*β* = −0.660, 95% CI −0.948 to −0.373, *P* < 0.001).

**Table 3:** Associations of hepatic steatosis with insulin resistance/sensitivity indices and circulating IGF-1 concentrations.

Outcome	Model	Ultrasound-defined MASLD		CAP, Continuous		
		St.β [95% CI], Yes or No	*P*-value	β [95% CI], per 10 dB/m	St.β [95% CI], per 1-SD	*P*-value
IGF-1	Model 1	-0.629 [−0.934, −0.325]	<0.001	-0.034 [−0.050, −0.018]	-0.264 [−0.390, −0.138]	<0.001
	Model 2	-0.420 [−0.663, −0.176]	<0.001	-0.022 [−0.036, −0.007]	-0.167 [−0.277, −0.058]	0.003
	Model 3	-0.451 [−0.693, −0.208]	<0.001	-0.022 [−0.036, −0.008]	-0.172 [−0.280, −0.064]	0.002
TG/HDL-C	Model 1	0.867 [0.572, 1.161]	<0.001	0.067 [0.045, 0.088]	0.373 [0.253, 0.494]	<0.001
	Model 2	0.786 [0.496, 1.076]	<0.001	0.056 [0.033, 0.080]	0.316 [0.185, 0.447]	<0.001
	Model 3	0.859 [0.574, 1.145]	<0.001	0.058 [0.035, 0.081]	0.322 [0.193, 0.451]	<0.001
TyG	Model 1	0.631 [0.327, 0.936]	<0.001	0.007 [0.004, 0.010]	0.274 [0.149, 0.399]	<0.001
	Model 2	0.623 [0.308, 0.938]	<0.001	0.006 [0.003, 0.010]	0.251 [0.109, 0.393]	<0.001
	Model 3	0.694 [0.386, 1.002]	<0.001	0.007 [0.003, 0.010]	0.259 [0.122, 0.397]	<0.001
eGDR	Model 1	-0.537 [−0.845, −0.229]	<0.001	-0.192 [−0.272, −0.112]	-0.300 [−0.424, −0.176]	<0.001
	Model 2	-0.357 [−0.566, −0.149]	<0.001	-0.083 [−0.143, −0.023]	-0.129 [−0.224, −0.035]	0.007
	Model 3	-0.348 [−0.561, −0.135]	0.001	-0.081 [−0.141, −0.020]	-0.126 [−0.221, −0.031]	0.009
ISI-Search	Model 1	-0.772 [−1.071, −0.473]	<0.001	-0.283 [−0.378, −0.189]	-0.365 [−0.486, −0.243]	<0.001
	Model 2	-0.650 [−0.934, −0.366]	<0.001	-0.235 [−0.333, −0.137]	-0.302 [−0.429, −0.176]	<0.001
	Model 3	-0.660 [−0.948, −0.373]	<0.001	-0.233 [−0.332, −0.135]	-0.300 [−0.427, −0.173]	<0.001

Model 1: Crude model (unadjusted).

Model 2: Adjusted for age, sex, diabetes duration, body mass index, average daily insulin dose per kilogram, fasting C-peptide, hypertension, lipid-lowering medication use, eGFR, current smoking status, and current alcohol use.

Model 3: Additionally adjusted for GADA, IA-2A, IAA, ICA, and ZnT8A.

Values are standardized β coefficients (St.β) with 95% confidence intervals (CIs), unless otherwise specified. For ultrasound-defined MASLD, MASLD status was retained as a binary exposure and continuous outcomes were standardized to z-scores; therefore, St.β represents the difference in outcome standard deviation units between participants with and without MASLD. For continuous CAP analyses, both unstandardized β coefficients per 10 dB/m increase in CAP and standardized β coefficients per 1-SD increase in CAP are presented. IGF-1, TG/HDL-C ratio, and TyG index were natural log-transformed before analysis. Higher TG/HDL-C and TyG values indicate greater insulin resistance, whereas higher eGDR and ISI-Search values indicate greater insulin sensitivity.

CAP, controlled attenuation parameter; eGDR, estimated glucose disposal rate; GADA, glutamic acid decarboxylase antibody; IAA, insulin autoantibody; IA-2A, protein tyrosine phosphatase-2 antibody; IGF-1, insulin-like growth factor-1; ISI-Search, insulin sensitivity index-Search; MASLD, metabolic dysfunction-associated steatotic liver disease; TG/HDL-C, triglyceride-to-high-density lipoprotein cholesterol ratio; TyG, triglyceride-glucose index; ZnT8A, zinc transporter 8 antibody.

Consistent associations were observed when hepatic fat burden was evaluated continuously using CAP. In fully adjusted analyses, each 10 dB/m increase in CAP was associated with lower ln (IGF-1) concentrations (*β* = −0.022, 95% CI −0.036 to −0.008, *P* = 0.002), higher ln (TG/HDL-C) ratios (*β* = 0.058, 95% CI 0.035–0.081, *P* < 0.001), and higher ln (TyG) indices (*β* = 0.007, 95% CI 0.003–0.010, *P* < 0.001). Higher CAP was also associated with lower eGDR (*β* = −0.081, 95% CI −0.141 to −0.020, *P* = 0.009) and lower ISI-Search values (*β* = −0.233, 95% CI −0.332 to −0.135, *P* < 0.001). These findings were consistent across both categorical and continuous assessments of hepatic steatosis, indicating concurrent associations of greater liver fat burden with lower circulating IGF-1 concentrations and greater insulin resistance in individuals with T1D. The overall pattern of associations remained robust after Benjamini–Hochberg FDR correction of the fully adjusted Model 3 analyses (Supplementary Table 5).

### 3.4 Association of circulating IGF-1 with indices of insulin resistance

Given the concurrent associations of hepatic steatosis with lower circulating IGF-1 concentrations and greater insulin resistance, we next examined the relationship between circulating IGF-1 and surrogate indices of insulin resistance (Table 4). In the fully adjusted Model 3, higher circulating IGF-1 concentrations were consistently associated with a more insulin-sensitive metabolic profile. Specifically, IGF-1 was inversely associated with the TG/HDL-C ratio (St.*β* = −0.281, 95% CI −0.443 to −0.119, *P* < 0.001) and TyG index (St.*β* = −0.286, 95% CI −0.456 to −0.117, *P* = 0.001), and positively associated with eGDR (St.*β* = 0.183, 95% CI 0.068–0.297, *P* = 0.002) and ISI-Search (St.*β* = 0.283, 95% CI 0.125–0.441, *P* < 0.001). These associations were generally consistent across the crude and sequentially adjusted models. All four associations remained statistically significant after Benjamini–Hochberg FDR correction in the fully adjusted analyses (all FDR-adjusted *P* ≤ 0.002; Supplementary Table 6). Collectively, these findings demonstrate a robust concurrent association between lower circulating IGF-1 concentrations and greater insulin resistance in individuals with T1D.

**Table 4:** Associations of circulating IGF-1 with insulin resistance/sensitivity indices.

Outcome	Model	IGF-1, St.β [95% CI]	*P*-value
TG/HDL-C	Model 1	-0.181 [−0.309, −0.052]	0.006
	Model 2	-0.295 [−0.455, −0.135]	<0.001
	Model 3	-0.281 [−0.443, −0.119]	<0.001
TyG	Model 1	-0.169 [−0.297, −0.041]	0.010
	Model 2	-0.305 [−0.474, −0.135]	<0.001
	Model 3	-0.286 [−0.456, −0.117]	0.001
eGDR	Model 1	0.205 [0.077, 0.332]	0.002
	Model 2	0.183 [0.072, 0.295]	0.001
	Model 3	0.183 [0.068, 0.297]	0.002
ISI-Search	Model 1	0.240 [0.114, 0.366]	<0.001
	Model 2	0.293 [0.139, 0.446]	<0.001
	Model 3	0.283 [0.125, 0.441]	<0.001

Model 1: Crude model (unadjusted).

Model 2: Adjusted for age, sex, diabetes duration, body mass index, average daily insulin dose per kilogram, fasting C-peptide, hypertension, lipid-lowering medication use, eGFR, current smoking status, and current alcohol use.

Model 3: Additionally adjusted for GADA, IA-2A, IAA, ICA, and ZnT8A.

Values are standardized β coefficients (St.β) with 95% confidence intervals (CIs) derived from linear regression models. Continuous exposure and outcome variables were standardized to z-scores before regression analysis. IGF-1, TG/HDL-C, and TyG index were natural log-transformed before analysis. Higher TG/HDL-C and TyG values indicate greater insulin resistance, whereas higher eGDR and ISI-Search values indicate greater insulin sensitivity.

eGDR, estimated glucose disposal rate; GADA, glutamic acid decarboxylase antibody; IAA, insulin autoantibody; IA-2A, protein tyrosine phosphatase-2 antibody; IGF-1, insulin-like growth factor-1; ISI-Search, insulin sensitivity index-Search; TG/HDL-C, triglyceride-to-high-density lipoprotein cholesterol ratio; TyG, triglyceride-glucose index; ZnT8A, zinc transporter 8 antibody.

### 3.5 Potential roles of IGF-1 and insulin resistance in the association between hepatic steatosis and short-term hyperglycemic excursions

To further explore the potential roles of circulating IGF-1 and insulin resistance in the association between hepatic steatosis and short-term glycemic dysregulation, serial mediation analyses were performed using 1,5-AG as the glycemic outcome. Given the cross-sectional design, these analyses were intended to quantify potential explanatory pathways rather than establish temporal or causal relationships. TG/HDL-C was selected as the primary surrogate measure of insulin resistance because it does not incorporate contemporaneous glucose concentrations in its calculation and is therefore less susceptible to mathematical coupling with the glycemic outcome.

In the primary model using TG/HDL-C (Figure 1A and Supplementary Table 7), ultrasound-defined MASLD was associated with lower 1,5-AG concentrations overall (total association: St.*β* = −0.475, 95% CI −0.795 to −0.155, *P* = 0.004). After inclusion of circulating IGF-1 and TG/HDL-C, the direct association was attenuated and was no longer statistically significant (St.*β* = −0.083, 95% CI −0.409–0.244, *P* = 0.618). In contrast, the total indirect association through IGF-1 and TG/HDL-C was statistically significant (St.*β* = −0.392, 95% CI −0.592 to −0.208), accounting for 82.6% of the total association. The specific indirect association through IGF-1 alone was −0.129 (95% CI −0.256 to −0.038), accounting for 27.1% of the total association, whereas that through TG/HDL-C alone was −0.239 (95% CI −0.402 to −0.090), accounting for 50.3%. A smaller but statistically significant serial indirect association involving both IGF-1 and TG/HDL-C was also observed (St.*β* = −0.025, 95% CI −0.066 to −0.001), accounting for 5.2% of the total association.

**Figure 1:**
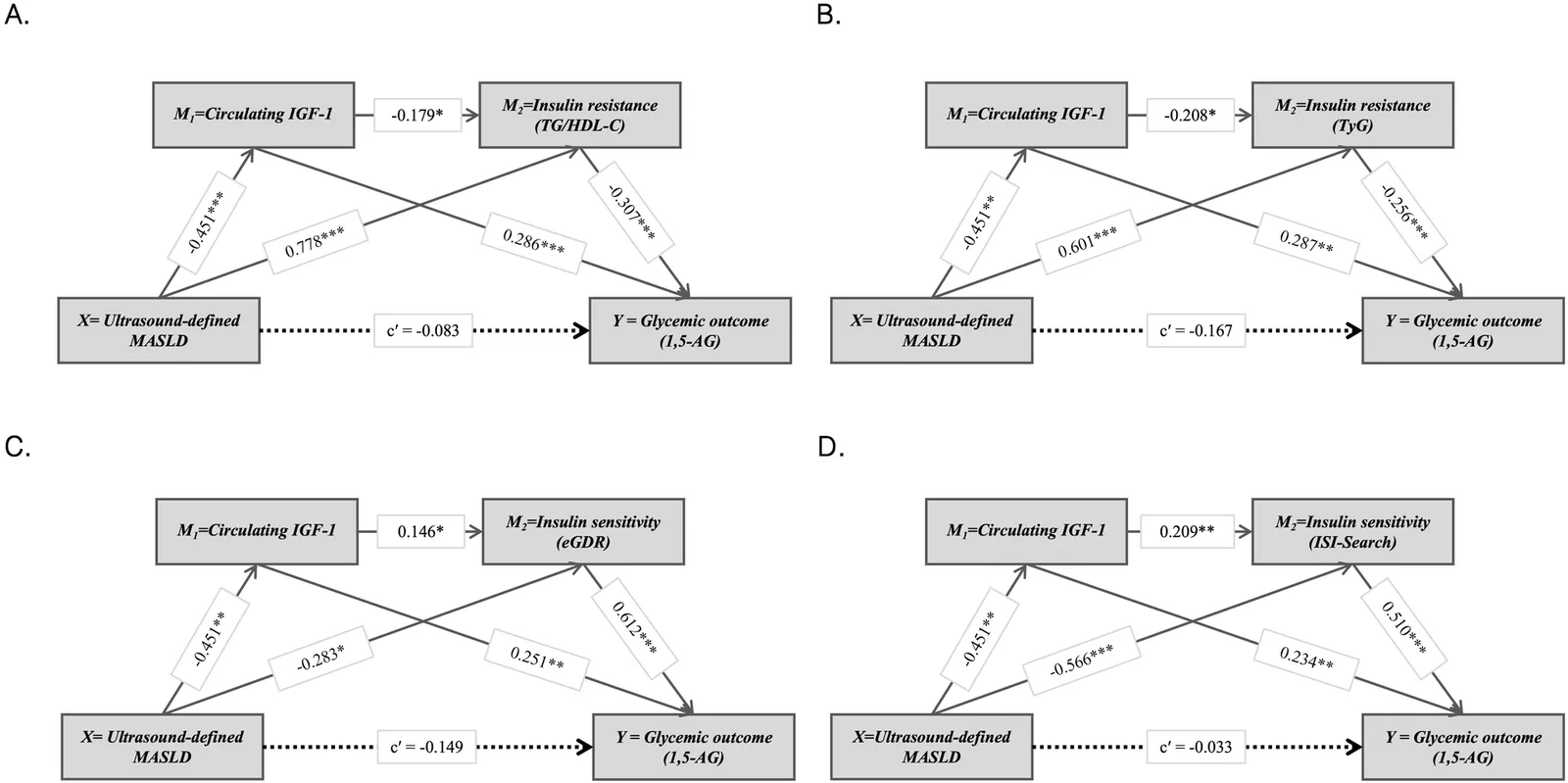
Serial mediation analysis of circulating IGF-1 and insulin resistance/sensitivity indices in the association between hepatic steatosis and short-term glycemic dysregulation. Serial mediation analyses were performed with ultrasound-defined MASLD as the exposure **(X)**, circulating IGF-1 as the first proposed mediator (M_1_), an insulin resistance or sensitivity index as the second proposed mediator (M_2_), and 1,5-AG as the glycemic outcome **(Y)**. TG/HDL-C was used as the primary measure of insulin resistance **(A)**, with TyG **(B)**, eGDR **(C)**, and ISI-Search **(D)** evaluated in sensitivity analyses. Higher TG/HDL-C and TyG values indicate greater insulin resistance, whereas higher eGDR and ISI-Search values indicate greater insulin sensitivity. Values shown along the paths are standardized regression coefficients. Solid arrows represent mediator-related paths, whereas the dashed arrow represents the direct association (c′) between MASLD and 1,5-AG after inclusion of the proposed mediators. Models were adjusted for age, sex, diabetes duration, body mass index, average daily insulin dose per kilogram, fasting C-peptide, hypertension, lipid-lowering medication use, eGFR, current smoking status, current alcohol use, GADA, IA-2A, IAA, ICA, and ZnT8A. Indirect associations and corresponding 95% bias-corrected bootstrap confidence intervals based on 5,000 bootstrap samples are presented in Supplementary Tables 7–10. *P < 0.05, **P < 0.01, ***P < 0.001.

Sensitivity analyses using TyG, eGDR, and ISI-Search as alternative surrogate measures of insulin resistance yielded consistent patterns (Figures 1B–D and Supplementary Tables 8–S10). The total indirect association remained statistically significant in all three sensitivity models, accounting for 64.7% of the total association in the TyG model, 68.7% in the eGDR model, and 93.1% in the ISI-Search model. The corresponding direct associations were not statistically significant in any of these models. Across the sensitivity analyses, the IGF-1-specific indirect association accounted for 22.2%–27.2% of the total association, the insulin resistance-specific indirect association for 32.4%–60.8%, and the serial pathway involving both IGF-1 and insulin resistance for 5.1%–10.1%.

Overall, these findings suggest that concurrent differences in circulating IGF-1 and insulin sensitivity may statistically account for a substantial proportion of the association between hepatic steatosis and lower 1,5-AG concentrations in individuals with T1D; however, the cross-sectional design precludes conclusions regarding temporal sequence or causality.

### 3.6 Sensitivity analyses

The robustness of the primary findings was further evaluated by alternatively defining MASLD using a CAP threshold of ≥238 dB/m. CAP-defined MASLD showed moderate agreement with ultrasound-defined MASLD, with an accuracy of 86.2% (95% CI 81.0%–90.3%), sensitivity of 70.0%, specificity of 90.6%, and *κ* = 0.597 (*P* < 0.001) (Supplementary Table 1^1^).

When MASLD was redefined using the CAP threshold, the overall pattern of associations remained consistent with the primary analyses (Supplementary Table 1^2^). In the fully adjusted model, CAP-defined MASLD remained significantly associated with lower 1,5-AG concentrations (St.*β* = −0.365, 95% CI −0.694 to −0.036, *P* = 0.030), lower circulating IGF-1 concentrations (St.*β* = −0.331, 95% CI −0.582 to −0.079, *P* = 0.010), and a more insulin-resistant metabolic profile. Consistent associations were also observed across major CGM-derived glycemic metrics, including higher PPG-2 h, glucose peak, MG, GMI, and TAR, together with lower TIR. Although a small number of secondary associations did not reach statistical significance after full adjustment, their directions were generally consistent with the primary analyses. Overall, these findings support the robustness of the principal associations across alternative definitions of hepatic steatosis.

## 4 Discussion

In this cross-sectional study of individuals with T1D, hepatic steatosis was independently associated with a distinct and less favorable glycemic phenotype, characterized particularly by abnormalities in short-term and postprandial glycemic measures. Ultrasound-defined MASLD and greater liver fat burden assessed by CAP were consistently associated with lower 1,5-AG concentrations, higher PPG-2 h, greater glucose peaks, higher MG and GMI, lower TIR, and higher TAR, whereas associations with fasting glucose were not significant. Individuals with hepatic steatosis also exhibited lower circulating IGF-1 concentrations and greater insulin resistance across multiple surrogate indices. These associations were largely preserved after multivariable adjustment and correction for multiple testing. Furthermore, serial mediation analyses suggested that concurrent differences in circulating IGF-1 and insulin sensitivity may statistically account for a substantial proportion of the association between hepatic steatosis and lower 1,5-AG concentrations. Together, these findings suggest that hepatic steatosis in T1D is associated with a metabolically distinct dysglycemic phenotype that may not be fully captured by fasting glucose alone.

The prevalence of ultrasound-defined MASLD in the present cohort was 21.6%, broadly consistent with previous estimates in T1D. A meta-analysis reported NAFLD in 19.3% of individuals with T1D overall and approximately 22% of adults, while another study using transient elastography and CAP reported a prevalence of approximately 20% (10). Considerable heterogeneity across previous studies has been attributed in part to differences in the methods used to assess hepatic steatosis (10). Despite these differences in prevalence, our findings extend a growing but heterogeneous body of evidence linking MASLD to adverse metabolic outcomes in T1D. Previous studies have also reported associations between hepatic steatosis and adverse glycemic characteristics in T1D; however, many relied on a single modality for liver fat assessment, a limited range of glycemic endpoints, or CGM measurements obtained under non-standardized conditions (12, 13, 35, 36). The present study extends this literature by combining ultrasonography with quantitative CAP assessment and integrating conventional glycemic markers, 1,5-AG, and CGM-derived metrics obtained under standardized inpatient conditions.

A notable finding of the present study was the stronger and more consistent association of hepatic steatosis with short-term and postprandial glycemic abnormalities than with fasting glycemia. The integration of 1,5-AG, which is particularly sensitive to recent hyperglycemic excursions and postprandial glucose exposure (37), with CGM-derived measures provided complementary evidence for this pattern. In particular, hepatic steatosis was associated with lower 1,5-AG concentrations, higher PPG-2 h and glucose peak, and less favorable TIR and TAR, whereas fasting glucose was not significantly associated with either ultrasound-defined MASLD or continuous CAP. These findings may be clinically relevant because HbA1c and fasting glucose alone do not fully characterize postprandial or short-term glycemic abnormalities. One potential explanation may involve altered regulation of hepatic glucose output after meals. In T1D, loss of endogenous amylin and insulin may contribute to impaired postprandial glucagon suppression, while altered hepatic glucagon signaling in steatotic liver disease could further influence hepatic glucose production and postprandial glucose excursions (38, 39). However, these mechanisms were not directly assessed in the present study and should therefore be considered speculative. Exploratory subgroup analyses identified several nominal interactions, although these findings should be interpreted cautiously and require further validation.

The concurrent associations of hepatic steatosis with circulating IGF-1 and insulin resistance provide a potential biological framework for the observed glycemic abnormalities. The liver is the principal source of circulating IGF-1, and hepatic steatosis has been associated with disturbances in GH signaling and hepatic IGF-1 production through mechanisms involving lipotoxicity, endoplasmic reticulum stress, oxidative stress, and altered growth hormone receptor signaling (21, 40, 41). In the present study, greater hepatic steatosis was associated with lower circulating IGF-1 concentrations and greater insulin resistance, while higher IGF-1 concentrations were consistently associated with a more insulin-sensitive profile. These observations are consistent with the insulin-sensitizing properties of IGF-1 (21, 42, 43), and support close interrelationships among hepatic steatosis, circulating IGF-1, and insulin sensitivity. Serial mediation analyses provided further statistical support for this framework: in the primary model, the combined indirect association through IGF-1 and insulin resistance accounted for 82.6% of the overall association between MASLD and 1,5-AG, with similar patterns observed using alternative insulin resistance indices. However, these analyses represent a statistical decomposition of concurrent associations rather than evidence of biological mediation or temporal sequence. Longitudinal and mechanistic studies are therefore needed to clarify the temporal relationships among hepatic steatosis, IGF-1, insulin sensitivity, and glycemic dysregulation.

Several potential clinical implications arise from these findings. Hepatic steatosis may help identify individuals with T1D who exhibit greater insulin resistance and less favorable glycemic profiles, consistent with previous observations linking hepatic steatosis in T1D to reduced insulin sensitivity and adverse metabolic characteristics (17–19). 1,5-AG and CGM may provide complementary information by capturing postprandial and daily glucose abnormalities that are not fully reflected by conventional glycemic measures, and recent T1D data have also linked MASLD with adverse CGM-derived metrics (35, 36). From a therapeutic perspective, lifestyle strategies targeting weight management, diet, and physical activity remain central to MASLD management (44). Whether reducing hepatic fat or improving insulin sensitivity can directly improve glycemic outcomes in T1D, however, requires prospective interventional investigation.

This study has several limitations. First, the cross-sectional design precludes conclusions regarding temporality or causality, and reverse or bidirectional relationships between hepatic steatosis and glycemic dysregulation remain possible. This limitation also applies to the serial mediation analyses. Second, CGM participation was voluntary, and participants who agreed to undergo CGM differed from those who declined participation in several metabolic characteristics. Although CAP and liver stiffness measurements were comparable between groups, some degree of selection bias related to voluntary CGM participation cannot be excluded. Third, insulin resistance was estimated using validated surrogate indices rather than the hyperinsulinemic-euglycemic clamp. Nevertheless, the consistency of the findings across four complementary indices supports the robustness of the observed associations. Fourth, ultrasonography has limited sensitivity and specificity for hepatic steatosis and may not completely distinguish steatosis from glycogenic hepatopathy in T1D; however, the consistent findings using continuous CAP and the CAP-based sensitivity definition provide additional support for the primary results. Finally, this was a single-center study conducted exclusively in Chinese individuals, which may limit the generalizability of the findings. Nevertheless, previous studies in European and U.S. T1D populations have reported similar associations of hepatic steatosis with insulin resistance and adverse metabolic characteristics (17–19), suggesting that these relationships may not be unique to the Chinese population. However, ethnic and regional differences in genetic background, body composition, dietary patterns, obesity prevalence, diabetes management, and healthcare systems may influence the magnitude of these associations. Multicenter studies in ethnically and geographically diverse T1D populations are therefore needed to confirm their broader applicability.

## 5 Conclusion

In individuals with T1D, hepatic steatosis was independently associated with a distinct dysglycemic phenotype characterized particularly by postprandial hyperglycemia, together with lower circulating IGF-1 concentrations and greater insulin resistance. Exploratory serial mediation analyses suggested that concurrent differences in circulating IGF-1 and insulin sensitivity may statistically account for a substantial proportion of the association between hepatic steatosis and short-term glycemic dysregulation. These findings highlight hepatic steatosis as a clinically relevant metabolic comorbidity in T1D and provide a potential framework linking liver fat burden, IGF-1, insulin resistance, and glycemic abnormalities. Prospective and mechanistic studies are needed to confirm these relationships and determine whether reducing hepatic fat or improving insulin sensitivity can favorably influence glycemic outcomes in T1D.

## Data Availability

The data supporting the findings of this study are not publicly available due to patient privacy and institutional restrictions but may be made available from the corresponding author upon reasonable request.

## References

[1] Wong VW-S Ekstedt M Wong GL-H Hagström H. Changing epidemiology, global trends and implications for outcomes of NAFLD. J Hepatol. (2023) 79(3):842–52. 10.1016/j.jhep.2023.04.036 37169151

[2] Younossi ZM Stepanova M Ong J Trimble G AlQahtani S Younossi I. Nonalcoholic steatohepatitis is the most rapidly increasing indication for liver transplantation in the United States. Clin Gastroenterol Hepatol. (2021) 19(3):580–589.e5. 10.1016/j.cgh.2020.05.064 32531342

[3] Targher G Corey KE Byrne CD Roden M. The complex link between NAFLD and type 2 diabetes mellitus—mechanisms and treatments. Nat Rev Gastroenterol Hepatol. (2021) 18(9):599–612. 10.1038/s41575-021-00448-y 33972770

[4] Memaj P Jornayvaz FR. Non-alcoholic fatty liver disease in type 1 diabetes: prevalence and pathophysiology. Front Endocrinol (Lausanne). (2022) 13:1031633. 10.3389/fendo.2022.1031633 36531463 PMC9752856

[5] Mertens J Van Gaal LF Francque SM De Block C. NAFLD in type 1 diabetes: overrated or underappreciated? Ther Adv Endocrinol Metab. (2021) 12:20420188211055557. 10.1177/20420188211055557 34840719 PMC8613893

[6] Bielka W Przezak A Molęda P Pius-Sadowska E Machaliński B. Double diabetes-when type 1 diabetes meets type 2 diabetes: definition, pathogenesis and recognition. Cardiovasc Diabetol. (2024) 23(1):62. 10.1186/s12933-024-02145-x 38341550 PMC10859035

[7] Van der Schueren B Ellis D Faradji RN Al-Ozairi E Rosen J Mathieu C. Obesity in people living with type 1 diabetes. Lancet Diabetes Endocrinol. (2021) 9(11):776–85. 10.1016/S2213-8587(21)00246-1 34600607

[8] Byrne CD Targher G. Non-alcoholic fatty liver disease-related risk of cardiovascular disease and other cardiac complications. Diabetes Obes Metab. (2022) 24(Suppl 2):28–43. 10.1111/dom.14484 34324263

[9] Mantovani A Morieri ML Aldigeri R Palmisano L Masulli M Bonomo K. MASLD, hepatic steatosis and fibrosis are associated with the prevalence of chronic kidney disease and retinopathy in adults with type 1 diabetes mellitus. Diabetes Metab. (2024) 50(1):101497. 10.1016/j.diabet.2023.101497 37992857

[10] de Vries M Westerink J Kaasjager KHAH De Valk HW. Prevalence of nonalcoholic fatty liver disease (*N*AFLD) in patients with type 1 diabetes mellitus: a systematic review and meta-analysis. J Clin Endocrinol Metab. (2020) 105(12):3842–53. 10.1210/clinem/dgaa575 32827432 PMC7526735

[11] Serdarova M Dimova R Chakarova N Grozeva G Todorova A Tsarkova P. Metabolic determinants of NAFLD in adults with type 1 diabetes. Diabetes Res Clin Pract. (2022) 186:109819. 10.1016/j.diabres.2022.109819 35248654

[12] Maffeis C Piona C Morandi A Marigliano M Morotti E Mancioppi V. Glycaemic control metrics and metabolic dysfunction-associated steatotic liver disease in children and adolescents with type 1 diabetes. Diabetes Obes Metab. (2024) 26(12):5896–905. 10.1111/dom.15961 39344839

[13] Vergani M Borella ND Rizzo M Conti M Perra S Bianconi E. Metabolic dysfunction-associated steatotic liver disease, insulin sensitivity and continuous glucose monitoring metrics in patients with type 1 diabetes: a multi-centre cross-sectional study. Diabetes Obes Metab. (2025) 27(6):3201–11. 10.1111/dom.16333 40083078 PMC12046442

[14] Ichikawa T Nakao K Hamasaki K Furukawa R Tsuruta S Ueda Y. Role of growth hormone, insulin-like growth factor 1 and insulin-like growth factor-binding protein 3 in development of non-alcoholic fatty liver disease. Hepatol Int. (2007) 1(2):287–94. 10.1007/s12072-007-9007-4 19669352 PMC2716823

[15] Schneider CV Gross S Balasubramani S Tomanová P Schrader C Fromme M. Insulin-like growth factor-1 reflects liver disease stage and improves prediction of liver-related mortality. Clin Gastroenterol Hepatol. (2025) 23(13):2559–69. 10.1016/j.cgh.2025.02.030 40378984

[16] Sumida Y Yonei Y Tanaka S Mori K Kanemasa K Imai S. Lower levels of insulin-like growth factor-1 standard deviation score are associated with histological severity of non-alcoholic fatty liver disease. Hepatol Res. (2015) 45(7):771–81. 10.1111/hepr.12408 25163357

[17] Grzelka-Woźniak A Uruska A Szymańska-Garbacz E Araszkiewicz A Jabłkowski M Czupryniak L. Indirect insulin resistance markers are associated with nonalcoholic fatty liver disease in type 1 diabetes. Pol Arch Intern Med. (2023) 133(5):16404. 10.20452/pamw.16404 36602856

[18] de Vries M Westerink J El-Morabit F Kaasjager HAH(K) De Valk HW. Prevalence of non-alcoholic fatty liver disease (*N*AFLD) and its association with surrogate markers of insulin resistance in patients with type 1 diabetes. Diabetes Res Clin Pract. (2022) 186:109827. 10.1016/j.diabres.2022.109827 35283265

[19] Taiwo A Merrill RA Wendt L Pape D Thakkar H Maschek JA. Metabolite perturbations in type 1 diabetes associated with metabolic dysfunction-associated steatotic liver disease. Front Endocrinol (Lausanne). (2025) 16:1500242. 10.3389/fendo.2025.1500242 40568565 PMC12188458

[20] Stanley TL Fourman LT Zheng I McClure CM Feldpausch MN Torriani M. Relationship of IGF-1 and IGF-binding proteins to disease severity and glycemia in nonalcoholic fatty liver disease. J Clin Endocrinol Metab. (2021) 106(2):e520–33. 10.1210/clinem/dgaa792 33125080 PMC7823253

[21] Jones JI Clemmons DR. Insulin-like growth factors and their binding proteins: biological actions. Endocr Rev. (1995) 16(1):3–34. 10.1210/edrv-16-1-3 7758431

[22] Addendum. 2. Classification and diagnosis of diabetes: standards of medical care in diabetes—2021. Diabetes care 2021;44(Suppl. 1):S15–S33. Diabetes Care. (2021) 44(9):2182. 10.2337/dc21-ad09 34135016

[23] Mu S McEvoy JW Ozkan B Tang O Christenson RH Selvin E. NT-proBNP reference intervals in healthy U.S. Children, Adolescents, and Adults. J Appl Lab Med. (2023) 8(4):700–12. 10.1093/jalm/jfad024 PMC10320012 37279581

[24] Levey AS Bosch JP Lewis JB Greene T Rogers N Roth D. A more accurate method to estimate glomerular filtration rate from serum creatinine: a new prediction equation. Modification of diet in renal disease study group. Ann Intern Med. (1999) 130(6):461–70. 10.7326/0003-4819-130-6-199903160-00002 10075613

[25] Chen S Meng G Wang X Jiang H Shen M Wang X. Analysis of frequency changes in CD8(+) regulatory T cell subsets in peripheral blood of individuals with type 1 diabetes. Diabetol Metab Syndr. (2024) 16(1):305. 10.1186/s13098-024-01549-9 39695694 PMC11656918

[26] Zhu M Xu K Chen Y Gu Y Zhang M Luo F. Identification of novel T1D risk loci and their association with age and islet function at diagnosis in autoantibody-positive T1D individuals: based on a two-stage genome-wide association study. Diabetes Care. (2019) 42(8):1414–21. 10.2337/dc18-2023 31152121

[27] Williams KV Erbey JR Becker D Arslanian S Orchard TJ. Can clinical factors estimate insulin resistance in type 1 diabetes? Diabetes. (2000) 49(4):626–32. 10.2337/diabetes.49.4.626 10871201

[28] Dabelea D D’Agostino RB Mason CC West N Hamman RF Mayer-Davis EJ. Development, validation and use of an insulin sensitivity score in youths with diabetes: the SEARCH for diabetes in youth study. Diabetologia. (2011) 54(1):78–86. 10.1007/s00125-010-1911-9 20886205 PMC5412950

[29] Tao L-C Xu J- Wang T- Hua F Li J-J. Triglyceride-glucose index as a marker in cardiovascular diseases: landscape and limitations. Cardiovasc Diabetol. (2022) 21(1):68. 10.1186/s12933-022-01511-x 35524263 PMC9078015

[30] Farrell GC Chitturi S Lau GKK Sollano JD. Guidelines for the assessment and management of non-alcoholic fatty liver disease in the Asia-Pacific region: executive summary. J Gastroenterol Hepatol. (2007) 22(6):775–7. 10.1111/j.1440-1746.2007.05002.x 17565629

[31] Baršić N. Overview and developments in noninvasive diagnosis of nonalcoholic fatty liver disease. World J Gastroenterol. (2012) 18(30):3945–54. 10.3748/wjg.v18.i30.3945 PMC3419991 22912545

[32] Rinella ME Neuschwander-Tetri BA Siddiqui MS Abdelmalek MF Caldwell S Barb D. AASLD practice guidance on the clinical assessment and management of nonalcoholic fatty liver disease. Hepatology. (2023) 77(5):1797–835. 10.1097/HEP.0000000000000323 36727674 PMC10735173

[33] Sasso M Beaugrand M De Ledinghen V Douvin C Marcellin P Poupon R. Controlled attenuation parameter (CAP): a novel VCTE™ guided ultrasonic attenuation measurement for the evaluation of hepatic steatosis: preliminary study and validation in a cohort of patients with chronic liver disease from various causes. Ultrasound Med Biol. (2010) 36(11):1825–35. 10.1016/j.ultrasmedbio.2010.07.005 20870345

[34] Huang Z Ng K Chen H Deng W Li Y. Validation of controlled attenuation parameter measured by FibroScan as a novel surrogate marker for the evaluation of metabolic derangement. Front Endocrinol (Lausanne). (2021) 12:739875. 10.3389/fendo.2021.739875 35173677 PMC8841525

[35] Aernouts C Beldé SPW Lambrechts J Mertens J Ledeganck KJ Francque SM. Metabolic dysfunction-associated steatotic liver disease is associated with worse time in ranges in type 1 diabetes. Diabetes Obes Metab. (2024) 26(9):3781–90. 10.1111/dom.15723 38924290

[36] Mantovani A Ciardullo S Sani E Csermely A Shtembari E Milani I. Association between continuous glucose monitoring metrics and metabolic dysfunction-associated steatotic liver disease in adults with type 1 diabetes undergoing vibration-controlled transient elastography: a multicenter cross-sectional study. Diabetes Metab. (2025) 51(5):101684. 10.1016/j.diabet.2025.101684 40623665

[37] Welter M Boritza KC Anghebem-Oliveira MI Henneberg R Hauser AB Rego FGM. Data for serum 1,5 anhydroglucitol concentration in different populations. Data Brief. (2018) 20:753–60. 10.1016/j.dib.2018.08.165 30211270 PMC6129696

[38] Fineman M Weyer C Maggs DG Strobel S Kolterman OG. The human amylin analog, pramlintide, reduces postprandial hyperglucagonemia in patients with type 2 diabetes mellitus. Horm Metab Res. (2002) 34(9):504–8. 10.1055/s-2002-34790 12384827

[39] Unger RH Orci L. Paracrinology of islets and the paracrinopathy of diabetes. Proc Natl Acad Sci U S A. (2010) 107(37):16009–12. 10.1073/pnas.1006639107 20798346 PMC2941311

[40] Arturi F Succurro E Procopio C Pedace E Mannino GC Lugarà M. Nonalcoholic fatty liver disease is associated with low circulating levels of insulin-like growth factor-I. J Clin Endocrinol Metab. (2011) 96(10):E1640–4. 10.1210/jc.2011-1227 21816784

[41] Ohkubo R Mu W-C Wang C-L Song Z Barthez M Wang Y. The hepatic integrated stress response suppresses the somatotroph axis to control liver damage in nonalcoholic fatty liver disease. Cell Rep. (2022) 41(11):111803. 10.1016/j.celrep.2022.111803 36516757 PMC9825120

[42] Liu Z Cordoba-Chacon J Kineman RD Cronstein BN Muzumdar R Gong Z. Growth hormone control of hepatic lipid metabolism. Diabetes. (2016) 65(12):3598–609. 10.2337/db16-0649 27679560 PMC5127251

[43] Nishizawa H Iguchi G Fukuoka H Takahashi M Suda K Bando H. IGF-I induces senescence of hepatic stellate cells and limits fibrosis in a p53-dependent manner. Sci Rep. (2016) 6:34605. 10.1038/srep34605 27721459 PMC5056388

[44] Tacke F Horn P Wai-Sun Wong V Ratziu V Bugianesi E Francque S. ASL-EASD-EASO clinical practice guidelines on the management of metabolic dysfunction-associated steatotic liver disease (MASLD). J Hepatol. (2024) 81(3):492–542. 10.1016/j.jhep.2024.04.031 38851997

