# Supplementary material for: Hepatic steatosis is associated with postprandial dysglycemia in type 1 diabetes: insights from dual-modality liver assessment
Source: Front Med (Lausanne). 2026 Oct 27;13:1933235. doi: 10.3389/fmed.2026.1933235 (PMC13748457; doi:10.3389/fmed.2026.1933235)
Supplement: Supplementary file 1 [file Datasheet1.pdf]

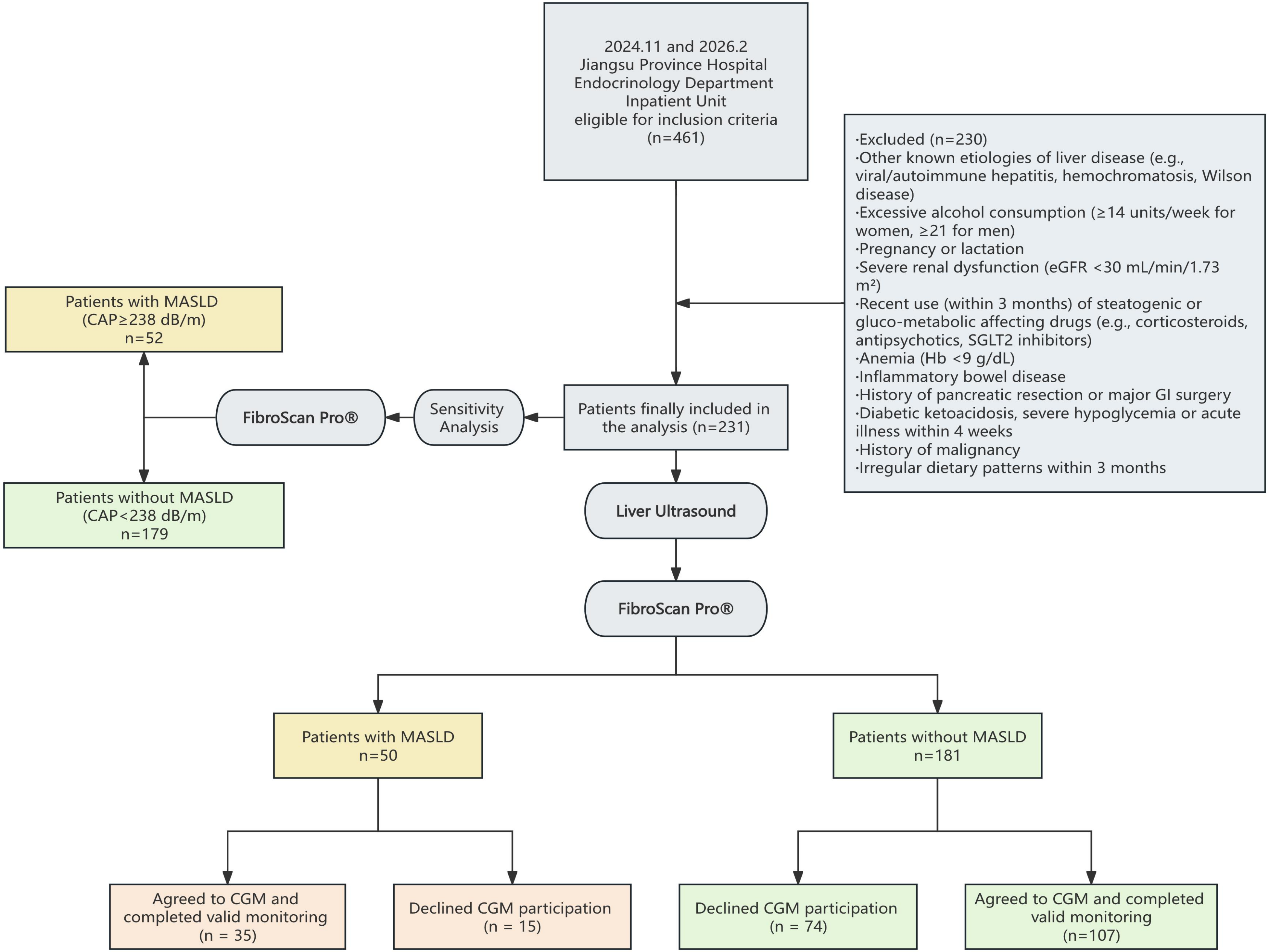
2024.11 and 2026.2
Jiangsu Province Hospital
Endocrinology Department
Inpatient Unit
eligible for inclusion criteria
(n=461)
·Excluded (n=230)
·Other known etiologies of liver disease (e.g., viral/autoimmune hepatitis, hemochromatosis, Wilson disease)
·Excessive alcohol consumption (≥14 units/week for women, ≥21 for men)
·Pregnancy or lactation
·Severe renal dysfunction (eGFR <30 mL/min/1.73 m²)
·Recent use (within 3 months) of steatogenic or gluco-metabolic affecting drugs (e.g., corticosteroids, antipsychotics, SGLT2 inhibitors)
·Anemia (Hb <9 g/dL)
·Inflammatory bowel disease
·History of pancreatic resection or major GI surgery
·Diabetic ketoacidosis, severe hypoglycemia or acute illness within 4 weeks
·History of malignancy
·Irregular dietary patterns within 3 months
Patients with MASLD (CAP≥238 dB/m) n=52
Patients without MASLD (CAP<238 dB/m) n=179
FibroScan Pro®
Sensitivity Analysis
Patients finally included in the analysis (n=231)
Liver Ultrasound
FibroScan Pro®
Patients with MASLD n=50
Patients without MASLD n=181
Agreed to CGM and completed valid monitoring (n = 35)
Declined CGM participation (n = 15)
Declined CGM participation (n = 74)
Agreed to CGM and completed valid monitoring (n=107)

Supplementary Figure 1. Flow diagram of study participant selection and assessments.

A total of 461 individuals with T1D were screened, of whom 230 were excluded according to the prespecified exclusion criteria, leaving 231 participants for the primary analyses. All included participants underwent liver ultrasonography and VCTE with CAP measurement. Ultrasound-defined MASLD was used as the primary categorical definition of hepatic steatosis, whereas CAP-defined MASLD (CAP ≥238 dB/m) was used in sensitivity analyses. CGM participation was voluntary; 142 participants agreed to undergo CGM and completed at least 72 hours of valid recording, whereas 89 declined CGM participation. The 142 participants with valid CGM data were included in CGM-based analyses.

Abbreviations: CAP, controlled attenuation parameter; CGM, continuous glucose monitoring; MASLD, metabolic dysfunction-associated steatotic liver disease; T1D, type 1 diabetes; VCTE, vibration-controlled transient elastography.

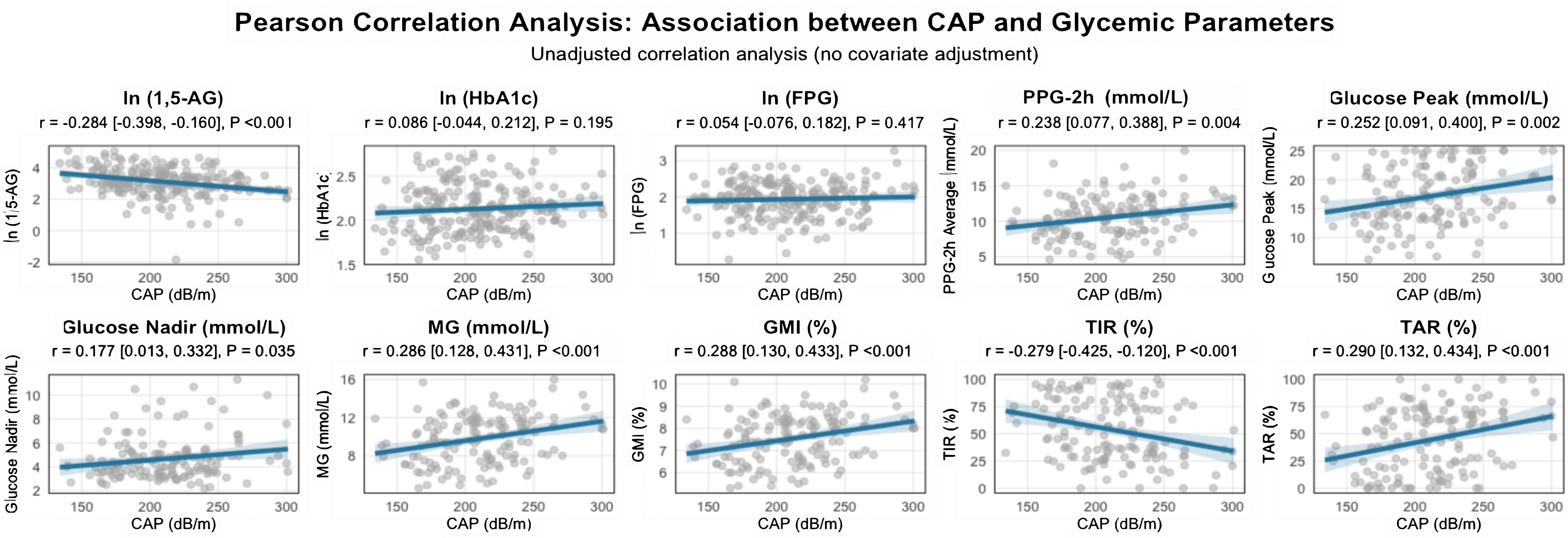
Pearson Correlation Analysis: Association between CAP and Glycemic Parameters
Unadjusted correlation analysis (no covariate adjustment)
ln (1,5-AG)
r = -0.284 [-0.398, -0.160], P <0.001
ln (HbA1c)
r = 0.086 [-0.044, 0.212], P = 0.195
ln (FPG)
r = 0.054 [-0.076, 0.182], P = 0.417
PPG-2h (mmol/L)
r = 0.238 [0.077, 0.388], P = 0.004
PPG-2h Average (mmol/L)
Glucose Peak (mmol/L)
r = 0.252 [0.091, 0.400], P = 0.002
Glucose Nadir (mmol/L)
r = 0.177 [0.013, 0.332], P = 0.035
MG (mmol/L)
r = 0.286 [0.128, 0.431], P <0.001
GMI (%)
r = 0.288 [0.130, 0.433], P <0.001
TIR (%)
r = -0.279 [-0.425, -0.120], P <0.001
TAR (%)
r = 0.290 [0.132, 0.434], P <0.001
CAP (dB/m)

Supplementary Figure 2. Correlations between CAP and glycemic parameters.

Scatterplots show the unadjusted Pearson correlations of CAP with biochemical and CGM-derived glycemic measures. Serum 1,5-AG, HbA1c, and fasting plasma glucose were natural log-transformed before analysis. Pearson correlation coefficients (r), corresponding 95% confidence intervals, and P values are shown in each panel. The solid line represents the linear regression fit, and the shaded area indicates the 95% confidence interval. Analyses of 1,5-AG, HbA1c, and fasting plasma glucose included 231 participants, whereas analyses of CGM-derived measures included the 142 participants with valid CGM data.

Abbreviations: 1,5-AG, 1,5-anhydroglucitol; CAP, controlled attenuation parameter; CGM, continuous glucose monitoring; FPG, fasting plasma glucose; GMI, glucose management indicator; MG, mean glucose; PPG-2h, 2-hour postprandial glucose; TAR, time above range; TIR, time in range

.

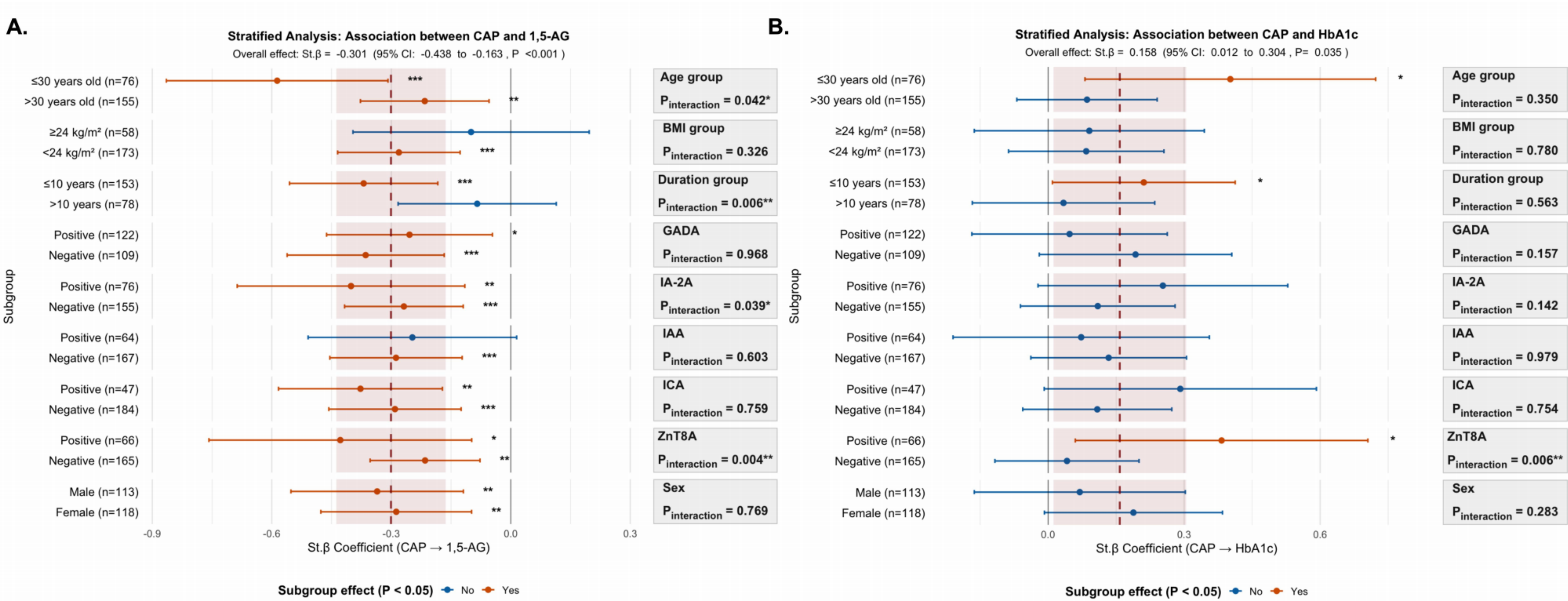

A.
Stratified Analysis: Association between CAP and 1,5-AG
Overall effect: St.β = -0.301 (95% CI: -0.438 to -0.163 , P <0.001)
≤30 years old (n=76)
>30 years old (n=155)
≥24 kg/m² (n=58)
<24 kg/m² (n=173)
≤10 years (n=153)
>10 years (n=78)
Positive (n=122)
Negative (n=109)
Positive (n=76)
Negative (n=155)
Positive (n=64)
Negative (n=167)
Positive (n=47)
Negative (n=184)
Positive (n=66)
Negative (n=165)
Male (n=113)
Female (n=118)
Age group P interaction = 0.042*
BMI group P interaction = 0.326
Duration group P interaction = 0.006**
GADA P interaction = 0.968
IA-2A P interaction = 0.039*
IAA P interaction = 0.603
ICA P interaction = 0.759
ZnT8A P interaction = 0.004**
Sex P interaction = 0.769
Subgroup
St.β Coefficient (CAP → 1,5-AG)
Subgroup effect (P < 0.05) No Yes
B.
Stratified Analysis: Association between CAP and HbA1c
Overall effect: St.β = 0.158 (95% CI: 0.012 to 0.304 , P= 0.035)
Age group P interaction = 0.350
BMI group P interaction = 0.780
Duration group P interaction = 0.563
GADA P interaction = 0.157
IA-2A P interaction = 0.142
IAA P interaction = 0.979
ICA P interaction = 0.754
ZnT8A P interaction = 0.006**
Sex P interaction = 0.283
St.β Coefficient (CAP → HbA1c)
Subgroup effect (P < 0.05) No Yes

Supplementary Figure 3. Stratified and interaction analyses of the associations of continuous CAP with 1,5-AG and HbA1c.

Forest plots show standardized β coefficients (St.β) and 95% confidence intervals for the associations of continuous CAP with ln-transformed 1,5-AG (A) and ln-transformed HbA1c (B) in the overall cohort and across prespecified subgroups. Models were adjusted for age, sex, diabetes duration , body mass index, average daily insulin dose per kilogram, fasting C-peptide, hypertension, lipid-lowering medication use, eGFR, current smoking status, current alcohol use, GADA, IA-2A, IAA, ICA, and ZnT8A, except for the variable used for stratification. P values for interaction were obtained from multiplicative interaction terms between continuous CAP and the corresponding subgroup variable in the fully adjusted models. These subgroup analyses were exploratory and should be interpreted cautiously.

Abbreviations: 1,5-AG, 1,5-anhydroglucitol; BMI, body mass index; CAP, controlled attenuation parameter; eGFR, estimated glomerular filtration rate; GADA, glutamic acid decarboxylase antibody; IAA, insulin autoantibody; IA-2A, protein tyrosine phosphatase-2 antibody; ICA, islet cell antibody; St.β, standardized beta coefficient; ZnT8A, zinc transporter 8 antibody.
