# Supplementary material for: Hepatic steatosis is associated with postprandial dysglycemia in type 1 diabetes: insights from dual-modality liver assessment
Source: Front Med (Lausanne). 2026 Oct 27;13:1933235. doi: 10.3389/fmed.2026.1933235 (PMC13748457; doi:10.3389/fmed.2026.1933235)
Supplement: Supplementary file 2 [file Table1.docx]

| **Supplementary Table 1. Baseline Characteristics According to CGM Participation** | | | | |
| --- | --- | --- | --- | --- |
| Variable | Overall (N = 231) | Declined CGM (n = 89) | Completed valid CGM (n = 142) | P value |
| **Demographics** |  |  |  |  |
| Age, years | 42.000 [23.833, 57.000] | 31.000 [18.000, 54.000] | 47.000 [33.000, 59.000] | <0.001 |
| Female sex, n (%) | 118 (51.1%) | 50 (56.2%) | 68 (47.9%) | 0.275 |
| **Anthropometrics** |  |  |  |  |
| BMI, kg/m² | 22.105 [20.200, 24.000] | 21.330 [19.977, 23.355] | 22.491 [20.477, 24.140] | 0.051 |
| Waist circumference, cm | 79.000 [72.000, 86.000] | 75.000 [71.000, 83.000] | 81.000 [73.000, 87.000] | <0.001 |
| Hypertension, n (%) | 52 (22.5%) | 14 (15.7%) | 38 (26.8%) | 0.073 |
| Diabetes characteristics |  |  |  |  |
| Age at diagnosis, years | 33.001 [16.923, 49.491] | 21.999 [12.500, 37.000] | 37.999 [22.000, 50.999] | <0.001 |
| Diabetes duration, years | 5.002 [1.224, 12.003] | 5.418 [2.000, 12.000] | 5.002 [0.999, 12.923] | 0.482 |
| Daily insulin dose, IU/kg | 0.550 [0.345, 0.698] | 0.564 [0.411, 0.737] | 0.550 [0.233, 0.667] | 0.059 |
| **Lifestyle factors** |  |  |  |  |
| Current smoker, n (%) | 47 (20.3%) | 15 (16.9%) | 32 (22.5%) | 0.381 |
| Current drinker, n (%) | 35 (15.2%) | 8 (9.0%) | 27 (19.0%) | 0.060 |
| Liver and renal parameters |  |  |  |  |
| CAP, dB/m | 208.000 [180.000, 232.000] | 204.000 [178.000, 228.000] | 209.000 [184.000, 236.000] | 0.211 |
| Liver stiffness, kPa | 4.600 [3.900, 5.600] | 4.600 [3.900, 5.500] | 4.600 [3.900, 5.600] | 0.889 |
| ALT, U/L | 15.000 [11.000, 21.000] | 13.000 [10.000, 16.000] | 16.000 [12.000, 22.000] | 0.005 |
| AST, U/L | 19.000 [16.000, 24.000] | 18.000 [16.000, 23.000] | 19.000 [16.000, 24.000] | 0.568 |
| eGFR, mL/min/1.73m² | 119.455 [99.639, 145.575] | 122.427 [101.972, 149.242] | 117.301 [97.108, 142.113] | 0.318 |
| **Lipid Profile** |  |  |  |  |
| Total cholesterol, mmol/L | 4.400 [3.720, 5.080] | 4.300 [3.720, 4.970] | 4.470 [3.720, 5.080] | 0.579 |
| Triglycerides, mmol/L | 0.890 [0.620, 1.250] | 0.770 [0.580, 1.020] | 0.990 [0.670, 1.490] | <0.001 |
| LDL cholesterol, mmol/L | 2.590 [2.130, 3.150] | 2.520 [2.120, 3.030] | 2.675 [2.150, 3.200] | 0.171 |
| HDL cholesterol, mmol/L | 1.300 [1.090, 1.530] | 1.370 [1.230, 1.560] | 1.200 [1.000, 1.460] | <0.001 |
| Lipid-lowering medication use, n (%) | 23 (10.0%) | 8 (9.0%) | 15 (10.6%) | 0.870 |
| **Insulin Resistance Indices** |  |  |  |  |
| eGDR | 8.736 [6.968, 10.202] | 9.688 [7.915, 10.929] | 8.326 [6.446, 9.495] | <0.001 |
| ISI-Search | 7.147 [5.529, 9.210] | 9.029 [6.626, 10.763] | 6.719 [5.134, 8.346] | <0.001 |
| TyG | 8.523 [8.039, 9.030] | 8.304 [7.884, 8.660] | 8.703 [8.218, 9.187] | <0.001 |
| TG/HDL-C | 1.625 [0.986, 2.502] | 1.257 [0.899, 1.709] | 1.836 [1.150, 3.073] | <0.001 |
| **β-cell Function** |  |  |  |  |
| Fasting C-peptide, pmol/L | 57.600 [2.355, 244.800] | 15.900 [2.355, 146.900] | 111.850 [2.355, 281.000] | 0.002 |
| **Autoantibodies** |  |  |  |  |
| IAA positive, n (%) | 64 (27.7%) | 34 (38.2%) | 30 (21.1%) | 0.008 |
| ICA positive, n (%) | 47 (20.3%) | 17 (19.1%) | 30 (21.1%) | 0.838 |
| IA-2A positive, n (%) | 76 (32.9%) | 39 (43.8%) | 37 (26.1%) | 0.008 |
| ZnT8A positive, n (%) | 66 (28.6%) | 30 (33.7%) | 36 (25.4%) | 0.223 |
| GADA positive, n (%) | 122 (52.8%) | 52 (58.4%) | 70 (49.3%) | 0.223 |
| **Glycemic Control** |  |  |  |  |
| HbA1c, % | 8.300 [6.790, 10.000] | 7.350 [6.460, 8.690] | 8.780 [7.360, 10.400] | <0.001 |
| Fasting glucose, mmol/L | 7.400 [5.300, 9.410] | 6.710 [5.080, 8.370] | 7.565 [5.480, 9.930] | 0.073 |
| 1,5-AG, μmol/L | 22.230 [14.450, 37.870] | 29.460 [19.680, 48.050] | 19.220 [13.180, 32.290] | <0.001 |
| Circulating IGF-1 |  |  |  |  |
| IGF-1, ng/mL | 125.000 [88.200, 164.000] | 142.000 [101.000, 176.000] | 116.500 [86.700, 149.000] | 0.005 |
| Note: Data are presented as median [Q1, Q3] for continuous variables and n (%) for categorical variables. P values were calculated using the Wilcoxon rank-sum test for continuous variables and the chi-square test or Fisher’s exact test, as appropriate, for categorical variables.  Abbreviations: 1,5-AG, 1,5-anhydroglucitol; ALT, alanine aminotransferase; AST, aspartate aminotransferase; BMI, body mass index; CAP, controlled attenuation parameter; CGM, continuous glucose monitoring; eGDR, estimated glucose disposal rate; eGFR, estimated glomerular filtration rate; GADA, glutamic acid decarboxylase antibody; HbA1c, glycated hemoglobin; HDL-C, high-density lipoprotein cholesterol; IAA, insulin autoantibody; IA-2A, protein tyrosine phosphatase-2 antibody; IGF-1, insulin-like growth factor-1; ISI-Search, insulin sensitivity index-Search; LDL-C, low-density lipoprotein cholesterol; TG/HDL-C, triglyceride-to-high-density lipoprotein cholesterol ratio; TyG, triglyceride-glucose index; ZnT8A, zinc transporter 8 antibody. | | | | |

| **Supplementary Table 2. False Discovery Rate-adjusted Associations between Hepatic Steatosis and Glycemic Profiles in the Fully Adjusted Model** | | | | | | | | |
| --- | --- | --- | --- | --- | --- | --- | --- | --- |
| **Outcome** | **Model** | **Ultrasound-defined MASLD** | | | **CAP, Continuous** | | | |
| **Venous Blood (n=231)** | | | | | | | | |
|  | | **St.β [95% CI],**  **Yes or No** | **P value** | **FDR-adjusted**  **P value** | **β [95% CI],**  **per 10 dB/m** | **St.β [95% CI], per 1-SD** | **P value** | **FDR-adjusted**  **P value** |
| 1,5-AG | Model 3 | -0.475 [-0.795, -0.155] | 0.004 | 0.005 | -0.076 [-0.110, -0.041] | -0.301 [-0.438, -0.163] | <0.001 | <0.001 |
| HbA1c | Model 3 | 0.467 [0.136, 0.797] | 0.006 | 0.007 | 0.012 [0.001, 0.023] | 0.158 [0.012, 0.304] | 0.035 | 0.039 |
| Fasting Glucose | Model 3 | 0.249 [-0.082, 0.581] | 0.139 | 0.155 | 0.007 [-0.012, 0.026] | 0.056 [-0.091, 0.202] | 0.456 | 0.456 |
| **CGM profiles (n=142)** | | | | | | | | |
| PPG-2h | Model 3 | 0.714 [0.331, 1.096] | <0.001 | 0.005 | 0.189 [0.033, 0.345] | 0.230 [0.040, 0.421] | 0.018 | 0.026 |
| Glucose Peak | Model 3 | 0.609 [0.210, 1.009] | 0.003 | 0.005 | 0.355 [0.077, 0.633] | 0.248 [0.054, 0.442] | 0.013 | 0.021 |
| Glucose Nadir | Model 3 | 0.270 [-0.118, 0.657] | 0.171 | 0.171 | 0.103 [0.008, 0.199] | 0.200 [0.016, 0.384] | 0.034 | 0.039 |
| MG | Model 3 | 0.709 [0.327, 1.092] | <0.001 | 0.002 | 0.215 [0.083, 0.347] | 0.302 [0.116, 0.488] | 0.002 | 0.003 |
| GMI | Model 3 | 0.709 [0.327, 1.092] | <0.001 | 0.002 | 0.093 [0.036, 0.151] | 0.304 [0.118, 0.490] | 0.002 | 0.003 |
| TIR | Model 3 | -0.647 [-1.032, -0.263] | 0.001 | 0.003 | -2.414 [-3.884, -0.943] | -0.304 [-0.489, -0.119] | 0.001 | 0.003 |
| TAR | Model 3 | 0.656 [0.271, 1.041] | <0.001 | 0.003 | 2.658 [1.115, 4.200] | 0.318 [0.134, 0.503] | <0.001 | 0.003 |
| Note: Results are derived from the fully adjusted Model 3, which included age, sex, diabetes duration, body mass index, average daily insulin dose per kilogram, fasting C-peptide, hypertension, lipid-lowering medication use, eGFR, current smoking status, current alcohol use, GADA, IA-2A, IAA, ICA, and ZnT8A. Values are presented as standardized β coefficients (St.β) with 95% confidence intervals (CIs). For continuous CAP analyses, unstandardized β coefficients per 10 dB/m increase in CAP are additionally reported. 1,5-AG, HbA1c, and fasting glucose were natural log-transformed before analysis. FDR-adjusted P values were calculated using the Benjamini–Hochberg procedure.  Abbreviations: 1,5-AG, 1,5-anhydroglucitol; CAP, controlled attenuation parameter; CGM, continuous glucose monitoring; GMI, glucose management indicator; MG, mean glucose; PPG-2h, 2-hour postprandial glucose; TAR, time above range; TIR, time in range. | | | | | | | | |

| **Supplementary Table 3. Stratified Analyses of the Association Between Continuous CAP and 1,5-AG** | | | | | |
| --- | --- | --- | --- | --- | --- |
| **Stratification Variable** | **Stratum Level** | **N** | **St.β [95% CI]** | **P value** | **P for interaction** |
| Overall | Overall | 231 | -0.301 [-0.438, -0.163] | <0.001 |  |
| Age group | >30 years old | 155 | -0.216 [-0.378, -0.054] | 0.010 | 0.042 |
|  | ≤30 years old | 76 | -0.587 [-0.865, -0.308] | <0.001 |  |
| BMI group | <24 kg/m² | 173 | -0.281 [-0.435, -0.127] | <0.001 | 0.326 |
|  | ≥24 kg/m² | 58 | -0.100 [-0.396, 0.197] | 0.514 |  |
| Duration group | >10 years | 78 | -0.084 [-0.283, 0.114] | 0.409 | 0.006 |
|  | ≤10 years | 153 | -0.369 [-0.556, -0.183] | <0.001 |  |
| GADA | Negative | 109 | -0.364 [-0.561, -0.167] | <0.001 | 0.968 |
|  | Positive | 122 | -0.254 [-0.462, -0.046] | 0.018 |  |
| IA-2A | Negative | 155 | -0.268 [-0.417, -0.119] | <0.001 | 0.039 |
|  | Positive | 76 | -0.401 [-0.687, -0.115] | 0.008 |  |
| IAA | Negative | 167 | -0.288 [-0.454, -0.122] | <0.001 | 0.603 |
|  | Positive | 64 | -0.247 [-0.509, 0.015] | 0.071 |  |
| ICA | Negative | 184 | -0.291 [-0.457, -0.125] | <0.001 | 0.759 |
|  | Positive | 47 | -0.377 [-0.583, -0.172] | 0.001 |  |
| ZnT8A | Negative | 165 | -0.215 [-0.353, -0.077] | 0.003 | 0.004 |
|  | Positive | 66 | -0.428 [-0.758, -0.098] | 0.014 |  |
| Sex | Female | 118 | -0.288 [-0.477, -0.098] | 0.004 | 0.769 |
|  | Male | 113 | -0.336 [-0.552, -0.119] | 0.003 |  |
| Note: Values are standardized β coefficients (St.β) with 95% confidence intervals (CIs) derived from stratified linear regression analyses. CAP and 1,5-AG were standardized to z-scores before regression analysis, and 1,5-AG was natural log-transformed before standardization. Models were adjusted for age, sex, diabetes duration, body mass index, average daily insulin dose per kilogram, fasting C-peptide, hypertension, lipid-lowering medication use, eGFR, current smoking status, current alcohol use, GADA, IA-2A, IAA, ICA, and ZnT8A, except for the variable used for stratification.  Abbreviations: 1,5-AG, 1,5-anhydroglucitol; BMI, body mass index; CAP, controlled attenuation parameter; eGFR, estimated glomerular filtration rate; GADA, glutamic acid decarboxylase antibody; IAA, insulin autoantibody; IA-2A, protein tyrosine phosphatase-2 antibody; ZnT8A, zinc transporter 8 antibody. | | | | | |

| **Supplementary Table 4. Stratified Analyses of the Association Between Continuous CAP and HbA1c** | | | | | |
| --- | --- | --- | --- | --- | --- |
| **Stratification Variable** | **Stratum Level** | **N** | **St.β [95% CI]** | **P value** | **P for interaction** |
| Overall | Overall | 231 | 0.158 [0.012, 0.304] | 0.035 |  |
| Age group | >30 years old | 155 | 0.086 [-0.069, 0.24] | 0.279 | 0.350 |
|  | ≤30 years old | 76 | 0.402 [0.081, 0.722] | 0.017 |  |
| BMI group | <24 kg/m² | 173 | 0.084 [-0.087, 0.255] | 0.338 | 0.780 |
|  | ≥24 kg/m² | 58 | 0.091 [-0.163, 0.344] | 0.487 |  |
| Duration group | >10 years | 78 | 0.034 [-0.167, 0.235] | 0.742 | 0.563 |
|  | ≤10 years | 153 | 0.211 [0.009, 0.413] | 0.042 |  |
| GADA | Negative | 109 | 0.193 [-0.020, 0.405] | 0.078 | 0.157 |
|  | Positive | 122 | 0.047 [-0.168, 0.263] | 0.668 |  |
| IA-2A | Negative | 155 | 0.110 [-0.061, 0.280] | 0.210 | 0.142 |
|  | Positive | 76 | 0.253 [-0.022, 0.529] | 0.077 |  |
| IAA | Negative | 167 | 0.134 [-0.038, 0.305] | 0.129 | 0.979 |
|  | Positive | 64 | 0.073 [-0.210, 0.356] | 0.616 |  |
| ICA | Negative | 184 | 0.108 [-0.056, 0.273] | 0.198 | 0.754 |
|  | Positive | 47 | 0.292 [-0.009, 0.592] | 0.067 |  |
| ZnT8A | Negative | 165 | 0.042 [-0.117, 0.200] | 0.609 | 0.006 |
|  | Positive | 66 | 0.383 [0.060, 0.705] | 0.024 |  |
| Sex | Female | 118 | 0.188 [-0.008, 0.384] | 0.063 | 0.283 |
|  | Male | 113 | 0.070 [-0.163, 0.303] | 0.558 |  |
| Note: Values are standardized β coefficients (St.β) with 95% confidence intervals (CIs) derived from stratified linear regression analyses. CAP and HbA1c were standardized to z-scores before regression analysis, and HbA1c was natural log-transformed before standardization. Models were adjusted for age, sex, diabetes duration, body mass index, average daily insulin dose per kilogram, fasting C-peptide, hypertension, lipid-lowering medication use, eGFR, current smoking status, current alcohol use, GADA, IA-2A, IAA, ICA, and ZnT8A, except for the variable used for stratification.  Abbreviations: HbA1c, glycated hemoglobin; BMI, body mass index; CAP, controlled attenuation parameter; eGFR, estimated glomerular filtration rate; GADA, glutamic acid decarboxylase antibody; IAA, insulin autoantibody; IA-2A, protein tyrosine phosphatase-2 antibody; ZnT8A, zinc transporter 8 antibody. | | | | | |

| **Supplementary Table 5. False Discovery Rate-adjusted Associations between Liver Steatosis and Insulin Resistance Indices in the Fully Adjusted Model** | | | | | | | | |
| --- | --- | --- | --- | --- | --- | --- | --- | --- |
| **Outcome** | **Model** | **Ultrasound-defined MASLD** | | | **CAP, Continuous** | | | |
|  |  | **St.β [95% CI]，**  **Yes or No** | **P value** | **FDR-adjusted P value** | **β [95% CI],**  **per 10 dB/m** | **St.β [95% CI],**  **per 1-SD** | **P value** | **FDR-adjusted P value** |
| TG/HDL-C | Model 3 | 0.859 [0.574, 1.145] | <0.001 | <0.001 | 0.058 [0.035, 0.081] | 0.322 [0.193, 0.451] | <0.001 | <0.001 |
| TyG | Model 3 | 0.694 [0.386, 1.002] | <0.001 | <0.001 | 0.007 [0.003, 0.010] | 0.259 [0.122, 0.397] | <0.001 | <0.001 |
| eGDR | Model 3 | -0.348 [-0.561, -0.135] | 0.001 | 0.002 | -0.081 [-0.141, -0.020] | -0.126 [-0.221, -0.031] | 0.009 | 0.009 |
| ISI-Search | Model 3 | -0.660 [-0.948, -0.373] | <0.001 | <0.001 | -0.233 [-0.332, -0.135] | -0.300 [-0.427, -0.173] | <0.001 | <0.001 |
| Results are derived from the fully adjusted Model 3, which included age, sex, diabetes duration, body mass index, average daily insulin dose per kilogram, fasting C-peptide, hypertension, lipid-lowering medication use, eGFR, current smoking status, current alcohol use, GADA, IA-2A, IAA, ICA, and ZnT8A. Values are presented as standardized β coefficients (St.β) with 95% confidence intervals (CIs). For continuous CAP analyses, unstandardized β coefficients per 10 dB/m increase in CAP are additionally reported. TG/HDL-C ratio and TyG index were natural log-transformed before analysis. FDR-adjusted P values were calculated using the Benjamini–Hochberg procedure.  Abbreviations: CAP, controlled attenuation parameter; TG/HDL-C, triglyceride-to-high-density lipoprotein cholesterol ratio; TyG, triglyceride-glucose index; eGDR, estimated glucose disposal rate. | | | | | | | | |

| **Supplementary Table 6. False Discovery Rate-adjusted Associations between Circulating IGF-1 and Insulin Resistance Indices in the Fully Adjusted Model** | | | | |
| --- | --- | --- | --- | --- |
| **Outcome** | **Model** | **IGF-1, St.β [95% CI]** | **P value** | **FDR-adjusted P value** |
| TG/HDL-C | Model 3 | -0.281 [-0.443, -0.119] | <0.001 | 0.001 |
| TyG | Model 3 | -0.286 [-0.456, -0.117] | 0.001 | 0.001 |
| eGDR | Model 3 | 0.183 [0.068, 0.297] | 0.002 | 0.002 |
| ISI-Search | Model 3 | 0.283 [0.125, 0.441] | <0.001 | 0.001 |
| Note: Results are derived from the fully adjusted Model 3, which included age, sex, diabetes duration, body mass index, average daily insulin dose per kilogram, fasting C-peptide, hypertension, lipid-lowering medication use, current smoking status, current alcohol use, eGFR, GADA, IA-2A, IAA, ICA, and ZnT8A. Values are presented as standardized β coefficients (St.β) with 95% confidence intervals (CIs). IGF-1, TG/HDL-C ratio, and TyG index were natural log-transformed before analysis. FDR-adjusted P values were calculated using the Benjamini–Hochberg procedure.  Abbreviations: TG/HDL-C, triglyceride-to-high-density lipoprotein cholesterol ratio; TyG, triglyceride-glucose index; eGDR, estimated glucose disposal rate; IGF-1, insulin-like growth factor-1. | | | | |

| **Supplementary Table 7. Serial Mediation Analysis: Association between Ultrasound-defined MASLD and 1,5-AG through IGF-1 and TG/HDL-C** | | | | |
| --- | --- | --- | --- | --- |
| **Effect Type** | **Unstandardized Effect [95% CI]** | **Standardized Effect [95% CI]** | **Proportion of total association, %** | **P value** |
| Total association (c) | -0.423 [-0.709, -0.138] | -0.475 [-0.795, -0.155] | 100.0 | 0.004 |
| Direct association (c′) | -0.074 [-0.365, 0.217] | -0.083 [-0.409, 0.244] |  | 0.618 |
| Total indirect association | -0.350 [-0.555, -0.173] | -0.392 [-0.592, -0.208] | 82.6 |  |
| **Specific indirect associations** |  |  |  |  |
| MASLD → IGF-1 → 1,5-AG | -0.115 [-0.230, -0.033] | -0.129 [-0.256, -0.038] | 27.1 |  |
| MASLD → TG/HDL-C → 1,5-AG | -0.213 [-0.379, -0.074] | -0.239 [-0.402, -0.090] | 50.3 |  |
| MASLD → IGF-1 → TG/HDL-C → 1,5-AG | -0.022 [-0.062, -0.0004] | -0.025 [-0.066, -0.0005] | 5.2 |  |
| Note: The model was adjusted for age, sex, diabetes duration, body mass index, average daily insulin dose per kilogram, fasting C-peptide, hypertension, lipid-lowering medication use, eGFR, current smoking status, current alcohol use, GADA, IA-2A, IAA, ICA, and ZnT8A. Indirect associations were considered statistically significant when the 95% bias-corrected bootstrap confidence interval did not include zero, based on 5,000 bootstrap samples. 1,5-AG, IGF-1, and TG/HDL-C ratio were natural log-transformed before analysis.  Abbreviations: MASLD, metabolic dysfunction-associated steatotic liver disease; IGF-1, insulin-like growth factor-1; TG/HDL-C, triglyceride-to-high-density lipoprotein cholesterol ratio; 1,5-AG, 1,5-anhydroglucitol; eGFR, estimated glomerular filtration rate. | | | | |

| **Supplementary Table 8. Serial Mediation Analysis: Association between Ultrasound-defined MASLD and 1,5-AG through IGF-1 and TyG** | | | | |
| --- | --- | --- | --- | --- |
| **Effect Type** | **Unstandardized Effect [95% CI]** | **Standardized Effect [95% CI]** | **Proportion of total association, %** | **P value** |
| Total association (c) | -0.423 [-0.709, -0.138] | -0.475 [-0.795, -0.155] | 100.0 | 0.004 |
| Direct association (c′) | -0.149 [-0.435, 0.136] | -0.167 [-0.487, 0.153] |  | 0.303 |
| Total indirect association | -0.274 [-0.457, -0.131] | -0.308 [-0.492, -0.152] | 64.7 |  |
| **Specific indirect associations** |  |  |  |  |
| MASLD → IGF-1 → 1,5-AG | -0.115 [-0.237, -0.032] | -0.129 [-0.255, -0.037] | 27.2 |  |
| MASLD → TyG → 1,5-AG | -0.137 [-0.270, -0.036] | -0.154 [-0.296, -0.042] | 32.4 |  |
| MASLD → IGF-1 → TyG → 1,5-AG | -0.021 [-0.064, -0.0005] | -0.024 [-0.070, -0.0006] | 5.1 |  |
| Note: The model was adjusted for age, sex, diabetes duration, body mass index, average daily insulin dose per kilogram, fasting C-peptide, hypertension, lipid-lowering medication use, eGFR, current smoking status, current alcohol use, GADA, IA-2A, IAA, ICA, and ZnT8A. Indirect associations were considered statistically significant when the 95% bias-corrected bootstrap confidence interval did not include zero, based on 5,000 bootstrap samples. 1,5-AG, IGF-1, and TyG were natural log-transformed before analysis.  Abbreviations: MASLD, metabolic dysfunction-associated steatotic liver disease; IGF-1, insulin-like growth factor-1; TyG, triglyceride-glucose index; 1,5-AG, 1,5-anhydroglucitol; eGFR, estimated glomerular filtration rate. | | | | |

| **Supplementary Table 9. Serial Mediation Analysis: Association between Ultrasound-defined MASLD and 1,5-AG through IGF-1 and eGDR** | | | | |
| --- | --- | --- | --- | --- |
| **Effect Type** | **Unstandardized Effect [95% CI]** | **Standardized Effect [95% CI]** | **Proportion of total association, %** | **P value** |
| Total association (c) | -0.423 [-0.709, -0.138] | -0.475 [-0.795, -0.155] | 100.0 | 0.004 |
| Direct association (c′) | -0.133 [-0.396, 0.131] | -0.149 [-0.444, 0.147] |  | 0.323 |
| Total indirect association | -0.291 [-0.513, -0.099] | -0.326 [-0.575, -0.111] | 68.7 |  |
| **Specific indirect associations** |  |  |  |  |
| MASLD → IGF-1 → 1,5-AG | -0.101 [-0.188, -0.034] | -0.113 [-0.211, -0.038] | 23.9 |  |
| MASLD → eGDR → 1,5-AG | -0.154 [-0.299, -0.036] | -0.173 [-0.336, -0.040] | 36.4 |  |
| MASLD → IGF-1 → eGDR → 1,5-AG | -0.036 [-0.089, -0.004] | -0.040 [-0.099, -0.004] | 8.4 |  |
| Note: The model was adjusted for age, sex, diabetes duration, body mass index, average daily insulin dose per kilogram, fasting C-peptide, hypertension, lipid-lowering medication use, eGFR, current smoking status, current alcohol use, GADA, IA-2A, IAA, ICA, and ZnT8A. Indirect associations were considered statistically significant when the 95% bias-corrected bootstrap confidence interval did not include zero, based on 5,000 bootstrap samples. 1,5-AG and IGF-1 were natural log-transformed before analysis. Abbreviations: MASLD, metabolic dysfunction-associated steatotic liver disease; IGF-1, insulin-like growth factor-1; eGDR, estimated glucose disposal rate; 1,5-AG, 1,5-anhydroglucitol; eGFR, estimated glomerular filtration rate. | | | | |

| **Supplementary Table 10. Serial Mediation Analysis: Association between Ultrasound-defined MASLD and 1,5-AG through IGF-1 and ISI-Search** | | | | |
| --- | --- | --- | --- | --- |
| **Effect Type** | **Unstandardized Effect [95% CI]** | **Standardized Effect [95% CI]** | **Proportion of total association, %** | **P value** |
| Total association (c) | -0.423 [-0.709, -0.138] | -0.475 [-0.795, -0.155] | 100.0 | 0.004 |
| Direct association (c′) | -0.029 [-0.290, 0.232] | -0.033 [-0.325, 0.260] |  | 0.826 |
| Total indirect association | -0.394 [-0.580, -0.226] | -0.442 [-0.640, -0.261] | 93.1 |  |
| **Specific indirect associations** |  |  |  |  |
| MASLD → IGF-1 → 1,5-AG | -0.094 [-0.193, -0.020] | -0.105 [-0.213, -0.023] | 22.2 |  |
| MASLD → ISI-Search → 1,5-AG | -0.258 [-0.400, -0.115] | -0.289 [-0.447, -0.132] | 60.8 |  |
| MASLD → IGF-1 → ISI-Search → 1,5-AG | -0.043 [-0.101, -0.008] | -0.048 [-0.111, -0.009] | 10.1 |  |
| Note: The model was adjusted for age, sex, diabetes duration, body mass index, average daily insulin dose per kilogram, fasting C-peptide, hypertension, lipid-lowering medication use, eGFR, current smoking status, current alcohol use, GADA, IA-2A, IAA, ICA, and ZnT8A. Indirect associations were considered statistically significant when the 95% bias-corrected bootstrap confidence interval did not include zero, based on 5,000 bootstrap samples. 1,5-AG and IGF-1 were natural log-transformed before analysis.  Abbreviations: MASLD, metabolic dysfunction-associated steatotic liver disease; IGF-1, insulin-like growth factor-1; ISI-Search, insulin sensitivity index-SEARCH; 1,5-AG, 1,5-anhydroglucitol; eGFR, estimated glomerular filtration rate. | | | | |

| **Supplementary Table 11. Agreement Between CAP-Defined and Ultrasound-Defined MASLD** | | | |
| --- | --- | --- | --- |
| **A. Cross-classification of CAP-defined and ultrasound-defined MASLD** | | | |
| Ultrasound-defined  CAP-defined | Positive | Negative | Total |
| **Positive** | 35 | 17 | 52 |
| **Negative** | 15 | 164 | 179 |
| Total | 50 | 181 | 231 |
| **B. Classification performance of CAP using ultrasound-defined MASLD as the reference** | | | |
| Measure | Estimate | | |
| Sensitivity | 70% | | |
| Specificity | 90.6% | | |
| Positive Predictive Value | 67.3% | | |
| Negative Predictive Value | 91.6% | | |
| Accuracy [95% CI] | 86.2% [81.0, 90.3] | | |
| Kappa [P value] | 0.597 [P<0.001] | | |
| Note: CAP-defined MASLD was defined as CAP ≥238 dB/m. Ultrasound-defined MASLD was used as the reference classification. Sensitivity was calculated as 35/50, specificity as 164/181, positive predictive value as 35/52, and negative predictive value as 164/179. Overall accuracy was calculated as (35 + 164)/231. Cohen’s κ was used to assess agreement beyond chance.  Abbreviations: CAP, controlled attenuation parameter; CI, confidence interval; MASLD, metabolic dysfunction-associated steatotic liver disease. | | | |

| **Supplementary Table 12. Associations of CAP-Defined MASLD with Glycemic Measures, Circulating IGF-1, and Insulin Resistance/Sensitivity Indices** | | | | |
| --- | --- | --- | --- | --- |
| **Outcome** | | **Model** | **CAP-defined MASLD (CAP ≥238 dB/m),**  **St.β [95% CI]** | **P value** |
| Glycemic control | 1,5-AG | Model 1 | -0.355 [-0.662, -0.047] | 0.024 |
|  |  | Model 2 | -0.352 [-0.677, -0.027] | 0.034 |
|  |  | Model 3 | -0.365 [-0.694, -0.036] | 0.030 |
|  | PPG-2h | Model 1 | 0.552 [0.173, 0.931] | 0.005 |
|  |  | Model 2 | 0.498 [0.105, 0.890] | 0.013 |
|  |  | Model 3 | 0.543 [0.141, 0.946] | 0.009 |
|  | Glucose peak | Model 1 | 0.532 [0.152, 0.912] | 0.006 |
|  |  | Model 2 | 0.485 [0.081, 0.888] | 0.019 |
|  |  | Model 3 | 0.512 [0.099, 0.924] | 0.015 |
|  | Glucose nadir | Model 1 | 0.359 [-0.026, 0.745] | 0.067 |
|  |  | Model 2 | 0.249 [-0.149, 0.647] | 0.218 |
|  |  | Model 3 | 0.324 [-0.071, 0.719] | 0.107 |
|  | MG | Model 1 | 0.611 [0.234, 0.988] | 0.002 |
|  |  | Model 2 | 0.553 [0.165, 0.941] | 0.006 |
|  |  | Model 3 | 0.609 [0.212, 1.006] | 0.003 |
|  | GMI | Model 1 | 0.620 [0.244, 0.996] | 0.001 |
|  |  | Model 2 | 0.563 [0.175, 0.951] | 0.005 |
|  |  | Model 3 | 0.620 [0.223, 1.016] | 0.002 |
|  | TIR | Model 1 | -0.609 [-0.986, -0.232] | 0.002 |
|  |  | Model 2 | -0.529 [-0.916, -0.142] | 0.008 |
|  |  | Model 3 | -0.563 [-0.960, -0.166] | 0.006 |
|  | TAR | Model 1 | 0.622 [0.246, 0.998] | 0.001 |
|  |  | Model 2 | 0.538 [0.149, 0.926] | 0.007 |
|  |  | Model 3 | 0.579 [0.181, 0.976] | 0.005 |
| Circulating IGF-1 | IGF-1 | Model 1 | -0.389 [-0.696, -0.083] | 0.013 |
|  |  | Model 2 | -0.259 [-0.512, -0.006] | 0.045 |
|  |  | Model 3 | -0.331 [-0.582, -0.079] | 0.010 |
| Insulin resistance/sensitivity indices | TG/HDL-C | Model 1 | 0.628 [0.328, 0.929] | <0.001 |
|  |  | Model 2 | 0.494 [0.186, 0.802] | 0.002 |
|  |  | Model 3 | 0.569 [0.264, 0.874] | <0.001 |
|  | **eGDR** | Model 1 | -0.448 [-0.754, -0.143] | 0.004 |
|  |  | Model 2 | -0.196 [-0.413, 0.020] | 0.076 |
|  |  | Model 3 | -0.215 [-0.435, 0.005] | 0.056 |
|  | TyG | Model 1 | 0.559 [0.257, 0.862] | <0.001 |
|  |  | Model 2 | 0.486 [0.160, 0.813] | 0.004 |
|  |  | Model 3 | 0.578 [0.258, 0.897] | <0.001 |
|  | ISI-Search | Model 1 | -0.558 [-0.860, -0.255] | <0.001 |
|  |  | Model 2 | -0.441 [-0.739, -0.144] | 0.004 |
|  |  | Model 3 | -0.489 [-0.790, -0.189] | 0.002 |
| Model 1: Crude model (unadjusted).  Model 2: Adjusted for age, sex, diabetes duration, body mass index, average daily insulin dose per kilogram, fasting C-peptide, hypertension, lipid-lowering medication use, eGFR, current smoking status, and current alcohol use.  Model 3: Additionally adjusted for GADA, IA-2A, IAA, ICA, and ZnT8A.  Note: Values are standardized β coefficients (St.β) with 95% confidence intervals (CIs) derived from linear regression models. CAP-defined MASLD was treated as a binary exposure, with CAP ≥238 dB/m indicating MASLD. Continuous outcomes were standardized to z-scores before regression analysis; therefore, St.β represents the difference in outcome standard deviation units between participants with and without CAP-defined MASLD.1,5-AG, IGF-1, TG/HDL-C ratio, and TyG index were natural log-transformed before analysis. Higher TG/HDL-C and TyG values indicate greater insulin resistance, whereas higher eGDR and ISI-Search values indicate greater insulin sensitivity.  Abbreviations: 1,5-AG, 1,5-anhydroglucitol; CAP, controlled attenuation parameter; CGM, continuous glucose monitoring; eGDR, estimated glucose disposal rate; GADA, glutamic acid decarboxylase antibody; GMI, glucose management indicator; IAA, insulin autoantibody; IA-2A, protein tyrosine phosphatase-2 antibody; IGF-1, insulin-like growth factor-1; ISI-Search, insulin sensitivity index-Search; MASLD, metabolic dysfunction-associated steatotic liver disease; MG, mean glucose; PPG-2h, 2-hour postprandial glucose; TAR, time above range; TG/HDL-C, triglyceride-to-high-density lipoprotein cholesterol ratio; TIR, time in range; TyG, triglyceride-glucose index; ZnT8A, zinc transporter 8 antibody. | | | | |
